# IgG4 drives M2a macrophages to a regulatory M2b‐like phenotype: potential implication in immune tolerance

**DOI:** 10.1111/all.13635

**Published:** 2018-11-28

**Authors:** Rodolfo Bianchini, Franziska Roth‐Walter, Anna Ohradanova‐Repic, Sabine Flicker, Karin Hufnagl, Michael Bernhard Fischer, Hannes Stockinger, Erika Jensen‐Jarolim

**Affiliations:** ^1^ Department of Comparative Medicine The Interuniversity Messerli Research Institute University of Veterinary Medicine Vienna Medical University of Vienna University Vienna Vienna Austria; ^2^ Institute for Hygiene and Applied Immunology Center for Pathophysiology, Infectiology and Immunology Medical University of Vienna Vienna Austria; ^3^ Institute of Pathophysiology and Allergy Research Center for Pathophysiology, Infectiology and Immunology Medical University of Vienna Vienna Austria; ^4^ Department of Blood Group Serology and Transfusion Medicine Medical University Vienna Vienna Austria; ^5^ Department of Health Science and Biomedicine Danube University Krems Vienna Austria

**Keywords:** allergy, CCL1, IgG4, immune tolerance, M2b macrophages

## Abstract

**Background:**

Macrophages can be converted *in vitro* into immunoregulatory M2b macrophages in the presence of immune complexes (ICs), but the role of the specific subclasses IgG1 or IgG4 in this phenotypic and functional change is not known.

**Objective:**

We aimed to refine the original method by applying precisely defined ICs of the subclasses IgG4 or IgG1 constructed by two independent methods.

**Methods:**

Monocyte‐derived macrophages (MDMs) were treated with M‐CSF, followed by IL‐4/IL‐13 to induce the M2a allergic phenotype. To mimic unspecific or allergen‐specific ICs, plates were coated with myeloma IgG1 or IgG4, or with grass pollen allergen Phl p 5 followed by recombinant human Phl p 5‐specific IgG1 or IgG4. M2a polarized macrophages were then added, cultured, and examined for cellular markers and cytokines by flow cytometry, ELISA, and rtPCR. Alternatively, immune complexes with IgG1 or IgG4 were formed using protein L.

**Results:**

IgG4 ICs down regulated CD163 and CD206 on M2a cells, and significantly increased IL‐10, IL‐6, TNFα, and CCL1 secretion, indicating a shift to an M2b‐like phenotype. Treatment with IgG4 ICs resulted in expression of FcγRII and down modulation of FcγRII compared with IgG1 treated cells (*P* = 0.0335) or untreated cells (*P* < 0.00001).

**Conclusion:**

Immune complexes with subclasses IgG1 and IgG4 can *in vitro* be generated by plate absorption, and in fluid form by protein L. Cross‐linking of FcγRIIb by the IgG4 subclass redirects pro‐allergic M2a macrophages to an M2b‐like immunosuppressive phenotype. This suggests an interplay of macrophages with IgG4 in immune tolerance, likely relevant in allergen immunotherapy.

**Abbreviations**
AIT: allergen‐specific immunotherapy
Breg/Bregs: B regulatory cell/s
CCL1: CC‐chemokine ligand 1
CCR8: C‐chemokine receptor 8
FcγR: Fc gamma receptor
IC: immune complex
IgG: immunoglobulin G
MDMs: monocyte‐derived macrophages
MFI: geometric mean of fluorescence intensity
rh‐: recombinant human
TNFα: tumor necrosis factor alpha
Treg/Tregs: T regulatory cell/s
αPhlp5‐IgG: anti‐Phl p 5 IgG

## 1 INTRODUCTION

In immediate‐type allergy, a particular Th2 cytokine microenvironment is established by inflammatory cytokines such as IL‐4, IL‐5, and IL‐13. These cytokines induce allergen‐specific IgE, eosinophilia, mucus production, and the recruitment of inflammatory cells to inflamed tissues.^1^, ^2^, ^3^ They polarize monocytes and macrophages into alternatively activated M2a macrophages typically characterized by downregulated expression of the hemoglobin‐haptoglobin scavenger receptor (CD163)^4^, ^5^ and upregulated expression of mannose receptor 1 (CD206) as well as of the B7‐2 costimulatory protein CD86. The CD86 upregulation is supported by basophil‐ and mast cell‐derived IL‐4 as shown for allergic pulmonary diseases.^6^ Moreover, these M2a macrophages secrete cytokines such as IL‐1 receptor antagonist (IL‐1ra)^7^ and chemokines such as the pulmonary and activation‐regulated chemokine (PARC; CCL18), the macrophage‐derived chemokine (MDC; CCL22), and the thymus and activation‐regulated chemokine (TARC; CCL17).^8^

Whereas the symptoms of allergic reactions can be combated by different pharmacological treatments, allergen immunotherapy (AIT) represents the only curative approach in type I allergy.^9^ AIT results in long‐term clinical benefits, and numerous publications highlight the induction of cellular responses within regulatory T cells (Treg), especially inducible IL‐10‐ and TGF‐β‐producing type 1 Treg (Tr1), and regulatory B cells (Bregs) as mechanisms of inducible tolerance.^9^, ^10^ Bregs are not only a source of IL‐10, but they also sense IL‐10 as a switching factor for IgG4 production.^11^ This is important as IgG4 is a hallmark of AIT, although its tolerogenic function is still insufficiently understood.^10^

While it is accepted that Tregs and Bregs with IL‐10 have a critical role in dampening the allergic inflammatory response in AIT, the role of macrophages in this process is not entirely defined. In particular, the M2b macrophages could take part in tolerance induction. M2b macrophages are specifically characterized by the secretion of CCL1 chemokine,^12^ a ligand of the cognate chemokine receptor CCR8.^13^, ^14^ Notably, CCR8 is not only essential for maintaining the M2b characteristics, it is also expressed by CCR8^+^FOXP3^+^ Treg cells.^15^ These master drivers of immune regulation could, therefore, be ignited by macrophages via CCL1 and CCR8.

Decisive for the present study were the observations that immune complexes (IC), without further specification, were compulsory to differentiate the M2b subtype from monocyte‐derived macrophages *in vitro*.^12^, ^16^ When recently subclass‐specific effects were addressed on M1 macrophages, IgG4 like IgG1 inhibited IFNγ signaling via FcγRI, favoring an M2‐like phenotype.^17^ When treating allergy, more or less the immunological opposite is desired, which is conversion from pro‐allergic M2a to M2b macrophages.

Our hypothesis combined all this background information considering the possibility to convert the pro‐allergic M2a subtype, highly present in the Th2 environment, into an immunoregulatory M2b‐like subtype. We (a) refined the original method, in which undefined immune complexes were applied for activating M2a macrophages, by using precisely defined immune complexes of subclasses IgG4 or IgG1; and (b) we applied two independent methods for the formation of such immune complexes, either plate bound or in solution. Subsequently, we screened for cellular markers and cytokines correlating with the induction of a tolerogenic microenvironment.

## 2 METHODS

### 2.1 Reagents and antibodies

All reagents and antibodies used in this study are reported in Tables S1‐S2.

### 2.2 Isolation and treatment of human monocytes

Blood drawing was approved by the institutional ethics committee of the Medical University of Vienna (ECS2007/2016), and healthy volunteers gave written informed consent for blood collection. The use of leukocyte reduction system chamber (LRS) as medical waste for a scientific purpose was approved by the institutional ethics committee of the Medical University of Vienna (ECS2177/2013). All experiments were conducted in accordance with the Helsinki Declaration of 1975 and followed institutional guidelines for good scientific practice (GSP).

Human peripheral blood was obtained either by venipuncture using vacuum tubes coated with lithium heparin (Greiner Bio‐One, Kremsmuenster, Austria) or by leukocyte reduction chambers (LRS) cones generated by the TrimaAccel automated blood collection system (Terumo BCT, Lakewood, CA, USA) during the process of single platelet apheresis. Peripheral blood mononuclear cells (PBMCs) were isolated from each donor using the cushion of Ficoll‐Paque (Data S1). Total PBMCs were seeded, and after 2 hours, the non adherent cells were washed away twice. The adherent cells (monocytes) were maintained in RPMI 1640 with 10% heat‐inactivated FBS, and 1% of P/S (cRPMI), supplemented with 20 ng/mL rh‐M‐CSF for 7‐9 days. Half of the medium was refreshed every 2‐3 days (Figure S1A‐B). The purity of monocytes was checked after 3 days, by flow cytometric analysis as CD3^−^CD11b^+^CD86^+^ adherent cells and determined as 80% ±5 of total live cells (Figure S2).

### 2.3 Macrophage polarization

After 7‐9 days, the cells acquired the mature macrophage characteristics. Macrophages were then removed using PBS^−Ca−Mg^ supplemented with 2.5 mmol/L EDTA (PBS/EDTA) pH 8.0 and allowed to recover in cRPMI. Then, the cells were polarized in M2o, M2a, M2b, and M2c as described in Table S3.

### 2.4 M2a stimulation with IgG1 or IgG4 immune complexes

#### 2.4.1 Creation of plate‐fixed immune complexes of IgG subclasses

To mimic immune complexes (ICs) consisting of human myeloma IgG1 (mIgG1) or IgG4 (mIgG4), a 96‐well plate (Falcon, Corning, NY, USA) was coated with these antibodies at 50 μg/mL in HBSS and washed twice with cRPMI (Data S1).

#### 2.4.2 Creation of soluble IgG1 and IgG4 immune complexes by protein L

To mimic soluble immune complexes (ICs) consisting of human myeloma IgG1 (mIgG1) or IgG4 (mIgG4), 7‐9 days differentiated monocytes were treated at room temperature for 30 minutes with mIgG1 or mIgG4 antibodies and then washed twice with cRPMI. The rProtein‐L (Thermo Fisher Scientific, Waltham, MA, USA) at a ratio of 1 : 4 (rProtein‐L : antibodies) was used to cross‐link the bound antibodies.^18^ (Data S1).

#### 2.4.3 Creation of allergen‐specific immune complexes

To mimic immune complexes with human anti‐Phl p 5 IgG1 (αPhlp5‐IgG1) or IgG4 (αPhlp5‐IgG4), a 96‐well plate (Falcon) was coated with 20 μg/mL of recombinant *Phleum pratense* grass pollen allergen Phl p 5 (rPhl p 5) (endotoxin content 0.003EU/μg) (Biomay, Vienna, Austria), and plates saturated with 0.01% of Tween‐20 in HBSS (T‐HBSS) supplemented with 3% BSA for 1 hour. Then, αPhlp5‐IgG1 or αPhlp5‐IgG4 antibodies were incubated at 50 μg/mL in HBSS for 1 hour, washed with T‐HBSS and once with cRPMI (Data S1).

#### 2.4.4 Cell culture on immune complexes

After washing IC‐coated plate, 1.5 × 10^5 ^cells/mL of detached MDMs were seeded, either on IC‐coated wells or control wells without coated antibodies and treated with rh‐M‐CSF (BioLegend, San Diego, CA, USA), rh‐IL‐4, and rh‐IL‐13 (ImmunoTools, Friesoythe, Germany) M2a cytokine mix (Figure S1A‐B).

### 2.5 ELISA

Supernatants from M2a cells incubated or not on IgG1‐ or IgG4‐IC‐coated plates were collected after 72 hours, and IL‐10, IL‐6, and TNFα were analyzed by ELISA (Thermo Fisher Scientific), IL‐12p70 by ELISA (BioLegend), and CCL1 by ELISA (R&D Systems, Minneapolis, MN, USA), following the supplier's instructions.

### 2.6 Staining and flow cytometric analysis

After 72 hours, cells incubated on IgG1‐ or IgG4‐IC‐coated plates were detached using ice‐cold PBS/EDTA and washed twice with HBSS plus 3% FBS as staining buffer, for surface marker phenotypization. Then, the cells were incubated with a multicolor staining mix of monoclonal antibodies against CD14, CD86, CD11b, CD163, and CD206 or their isotype controls (BioLegend) diluted 1:100 in staining buffer for 30 minutes at 4°C followed by 2× washing with staining buffer.

For the FcγR staining, the cells were detached as described above, washed with an ice‐cold staining buffer (PBS^−Ca−Mg^ plus 1% BSA, 0.02% NaN_3_), and then blocked with 2.4 mg/mL human IgG (Beriglobin P; CSL Behring, King of Prussia, PA, USA) in staining buffer for 30 minutes on ice. The cells were then incubated with a multicolor staining mix of primary monoclonal antibodies against CD64, CD32, and CD16 or isotype controls (as specified in Table S2; diluted 1:40‐1:80 with the staining buffer) at 4°C for 30 minutes and washed twice with the staining buffer. Samples were acquired by FACS Canto II or LRSII flow cytometers (Becton Dickinson, Franklin Lakes, NJ, USA). Recorded events were analyzed with the FlowJo software version 10.3 (FlowJo, LLC, Ashland, OR, USA), and geometric mean fluorescence intensity (MFI) values were calculated for each fluorochrome. The z‐normalization of MFI for each staining antibody and each donor was performed for M2a, M2a + IgG1, and M2a + IgG1 (Data S1).

### 2.7 RNA isolation and reverse transcription

Polarized macrophages were collected after 48 hours and the pellet lysed in TRIzol (Sigma‐Aldrich, St. Louis, MO, USA). Total RNA was isolated using Direct‐zol RNA MiniPrep column system (Zymo Research, Irvine, CA, USA) with DNase I digestion step (15 minutes, RT). The resulting total RNA was measured by NanoDrop (Implen, Munich, Germany). cDNA synthesis was performed with 200 ng RNA/sample using iScript cDNA Synthesis Kit according to manufacturer's recommendations (Bio‐Rad, Hercules, CA, USA).

### 2.8 Real‐time polymerase chain reaction

The primers (Table S4) were designed using Primer‐Blast tool^19^ and evaluated with Beacon Designer Free Edition (Premier Biosoft, Palo Alto, CA, USA). Real‐time PCR (rtPCR) was performed using the Solis Biodyne Supermix (Solis Biodyne, Tartu, Estonia) in accordance with manufacturer's recommendations. rtPCR was performed on a QuantStudio 12K Flex system (Thermo Fisher Scientific) (Data S1).

The 2^−ΔΔCt^ analysis was performed using the QuantStudio 12k Flex Software v1.2.3 (Thermo Fisher Scientific) to obtain the cycle of threshold (Ct) for each sample investigated. Actin beta (ACTB) was used as reference gene. The mean fold change expression was calculated for M2a, M2a + IgG1, and M2a + IgG4.

### 2.9 Graphs and statistical analysis

The graphs and the statistical analyses were performed with GraphPad Prism version 6.00 for Macintosh (GraphPad Software, La Jolla, CA, USA). Validation of data was done by repeated‐measures one‐way ANOVA and Tukey multiple comparison post‐hoc test. Student's *t* test was performed to compare the rtPCR values of M2a + IgG1 and M2a + IgG4. The level of statistical significance is defined as n.s *P* > 0.05 (not significant), *P < 0.05, **P < 0.01, ***P < 0.001, ****P < 0.0001.

## 3 RESULTS

### 3.1 Surface markers clearly distinguish M2a and M2b macrophages

**Figure 1:**
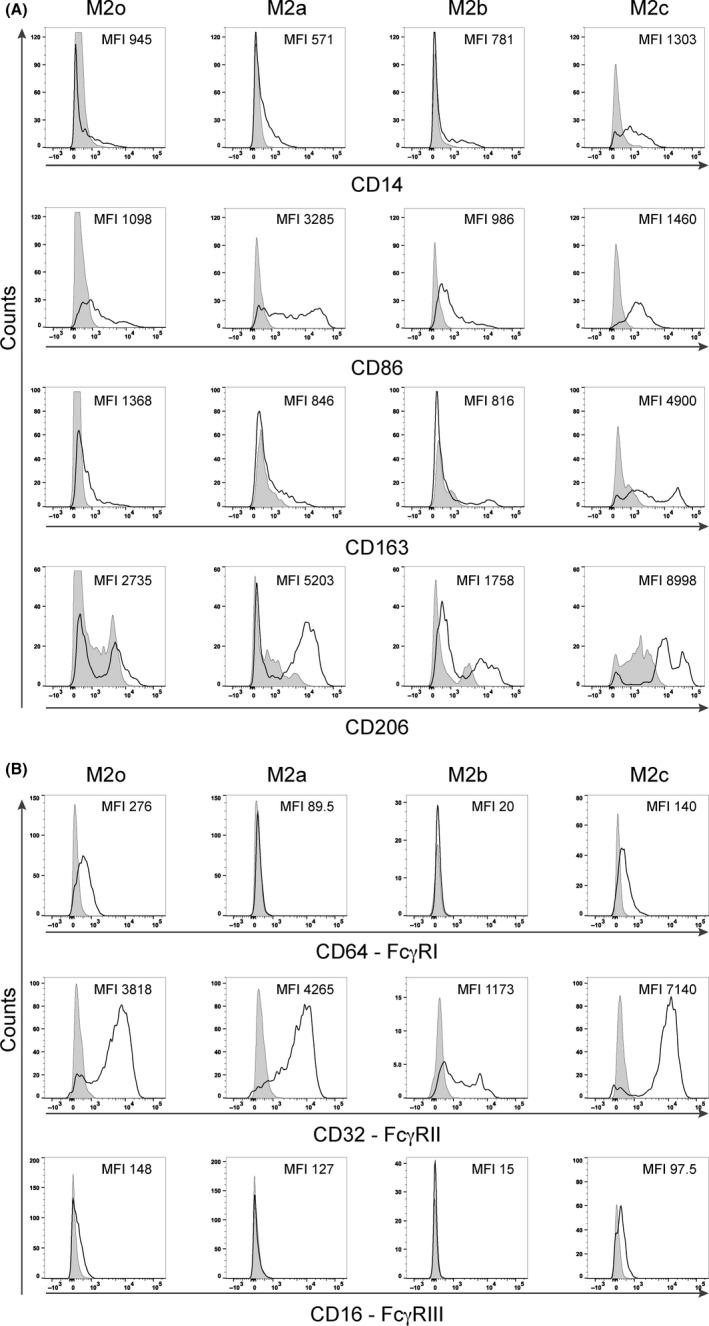
Flow cytometry phenotypizations of M2o, M2a, M2b, and M2c macrophages polarized *in vitro* for 72 h. A, Representative flow cytometry analysis of CD14, CD86, CD163, and CD206 surface marker expressions in one representative individual (nine donors in four independent experiments). B, Representative flow cytometry analysis of FcγR surface marker expressions in the same representative individual (six donors. in three independent experiments). The *y*‐axis represents the events normalized to the mode for each evaluated surface marker; empty curves: staining with specific antibodies, gray: isotype control. The MFI means of each marker are depicted within the graphs

The flow cytometric analyses of nine donors in four independent experiments were performed to distinguish between the different alternatively activated macrophages polarized *in vitro*. The analyses evaluated the surface marker expression of CD206, CD163, and the co stimulatory molecule B7‐2 (CD86), considered as specific markers of M2 alternatively activated population.^8^ The results showed that M2a macrophages had a higher expression of CD206 compared with M2o polarized macrophages, the CD163 expression was relatively higher in M2a macrophages than in M2b, but lower than in M2c, and the expression of CD86 was higher in M2a macrophages than in M2b or M2c (Figure 1A). Additionally, we analyzed the surface expression of high‐affinity Fc gamma receptors FcγRI‐CD64, low‐affinity FcγRII‐CD32, and low‐affinity FcγRIII‐CD16 of six different donors in three independent experiments. Apparently, the different M2o, M2a, M2b, M2c subpopulations differentially express Fc gamma receptors in relation to their functional status. M2o and M2c expressed FcγRI‐CD64 and FcγRIII‐CD16 (Figure 1B). High expression of the low‐affinity FcγRII‐CD32 was detected in all analyzed subpopulations, but this receptor was the most expressed Fcγ receptor in the M2a and M2b subpopulations in comparison with the other receptors (Figure 1B). All tested surface markers on various macrophage subpopulations are given as MFI ± standard deviation of the specific antibody used in the flow cytometry analysis (see Table S5A‐B).

### 3.2 Contrasting cytokine expression between M2a and M2c, and M2b

The method of choice to assess the differences between M2a pro‐allergic and M2b immunoregulatory macrophage subpopulation is their cytokine and chemokine patterns.^12^

**Figure 2:**
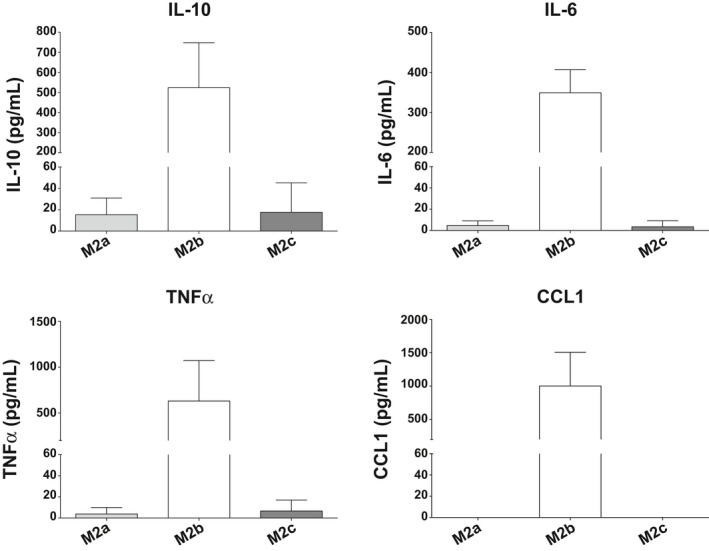
ELISA analyses of cytokine and chemokine secreted in the supernatant of M2a, M2b, and M2c macrophages polarized *in vitro* for 72 h. High levels of IL‐10, IL6, TNFα, and CCL1 are secreted by M2b macrophages (white bars), but not by M2a (light gray bars) or M2c macrophages (dark gray bars). The results from three independent experiments were combined for statistical analysis. Bars represent the mean concentration (pg/mL) of the measured cytokines ± STDEV

M2a and M2c macrophages slightly expressed IL‐10, but almost no IL‐6 and TNFα, and no CCL1 (Figure 2). In contrast, M2b macrophages secreted high levels of these cytokines and chemokines, with mean values of 524 and 1000 pg/mL for IL‐10 and CCL1, respectively, after 72 hours of polarization. No expression of the cytokine IL‐12p70 was detected in M2a, M2b, and M2c subpopulations (data not shown).

### 3.3 M2a macrophages stimulated with IgG4 develop an M2b‐like phenotype

**Figure 3:**
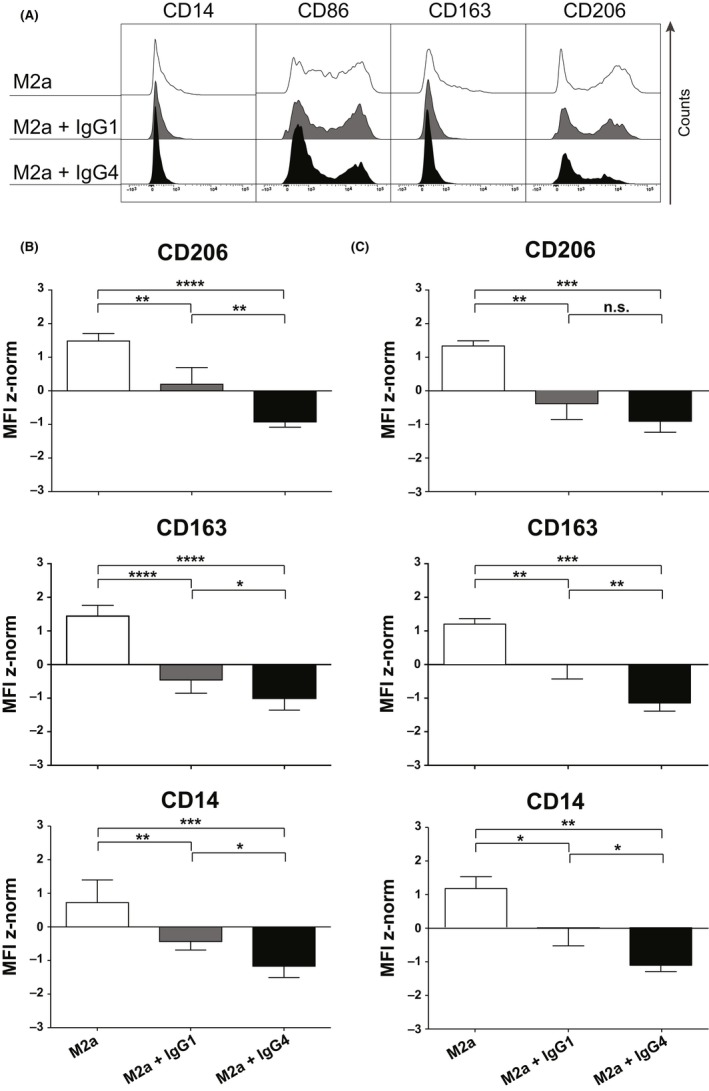
Flow cytometric analysis of surface markers expressed by M2a macrophages treated with myeloma (m)IgG1 or mIgG4 *in vitro* for 72 h. A, Half‐offset representation of specific M2a surface markers upon mIgG4 treatment of one representative donor; *y*‐axis: events normalized to the mode for each surface marker. Empty curves: staining of M2a cells; gray: staining of M2a treated with IgG1‐containing ICs (M2a + IgG1); black: staining of M2a with IgG4‐containing ICs (M2a + IgG4). B, Surface maker analysis using plate‐bound IC comprising IgG1 or IgG4 subclasses. C, Surface maker analysis using soluble IC treatment, formed by IgG1 or IgG4 subclasses followed by cross‐linking with rProtein‐L. MFI z‐normalization was performed for each marker and each donor before the statistical validation. White bars: M2a cells; gray: M2a cells + IgG1; dark: M2a cells + IgG4. The results from four independent experiments were combined for statistical analysis. Repeated‐measures one‐way ANOVA statistical analysis and Tukey multiple comparison post‐hoc test were performed, *P < 0.05, **P < 0.01, ***P < 0.001, ****P < 0.0001, n.s. not significant

M2a macrophages, treated with allergen nonspecific ICs containing mIgG1 or mIgG4, were subjected to surface marker expression analysis for the phenotypization. The results demonstrated that an M2b‐like phenotype was achieved when the M2a cells were treated with mIgG4 (Figure 3A,B). In fact, though both ICs with mIgG4 or with mIgG1 induced a significant reduction of CD163 expression in M2a cells (*P* < 0.0001) compared to unstimulated M2a controls, ICs with mIgG4 (*P* < 0.0001) were more competent than IgG1‐containing ICs (*P* = 0.0064) to further decrease CD206 expression. To confirm the relevance of our observations, we mimicked soluble immune complexes by an alternative approach. IgG1 or IgG4 were allowed to bind to the macrophage followed by rProtein‐L incubation to cross‐link their κ‐light chains. Also by this novel method, a significantly greater downmodulation of CD14, CD163, and CD206 surface markers was achieved by IgG4 than with IgG1 (Figure 3C), thus rendered results comparable with those obtained by plate‐bound immune complexes (Figure 3B). The CD86 surface expression did not vary among the differently treated M2a cells in both methods, though a trend in decreased CD86 expression upon M2a treatment with IgG4 ICs was observed (data not shown). The different expression patterns of all stained surface markers are summarized in Figure S3A. Taken together, the treatment of M2a with ICs consisting of IgG4, but not of IgG1, significantly promoted an M2b‐like phenotype (Figure 1).

### 3.4 Only M2a macrophages stimulated with IgG4 secrete M2b‐like cytokines

**Figure 4:**
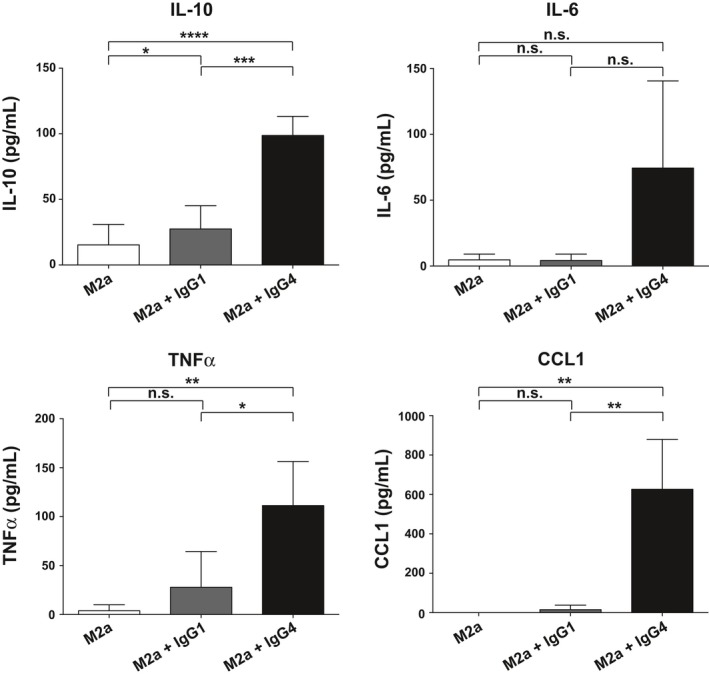
ELISA analyses of cytokine and chemokine secreted in the supernatant of M2a alone or treated with IgG1‐ (M2a + IgG1) or with IgG4‐containing ICs (M2a + IgG4) *in vitro* for 72 h. The results from three independent experiments were combined for statistical analysis. Repeated‐measures one‐way ANOVA statistical analysis and Tukey multiple comparison post‐hoc test were performed, *P < 0.05, **P < 0.01, ***P < 0.001, ****P < 0.0001, n.s. not significant

To investigate whether upon IgG4 stimulation, the M2a cells would not only phenotypically, but also functionally adapt an M2b‐like phenotype, secreted cytokines and chemokines were analyzed. The pattern of cytokine and chemokine expression by M2a, when stimulated with IgG4 containing ICs (Figure 4), resembled a pattern typical for M2b regulatory macrophages (Figure 2) and was different from IgG1 or no‐ICs stimulation for each tested cytokine. Levels of investigated cytokines and chemokine are given in Table S6. An almost exclusive secretion of CCL1 was observed when M2a macrophages were stimulated with ICs containing IgG4 than IgG1 compared with M2a alone (Table S6 and Figure S3C). Therefore, ICs formed with mIgG4 also functionally drive the polarization status of pro‐allergic M2a macrophages toward an M2b‐like regulatory subtype.

### 3.5 M2a macrophages stimulated with IgG4 reduce FcγRII surface expression but upregulate the mRNA of FCGRIIB gene

**Figure 5:**
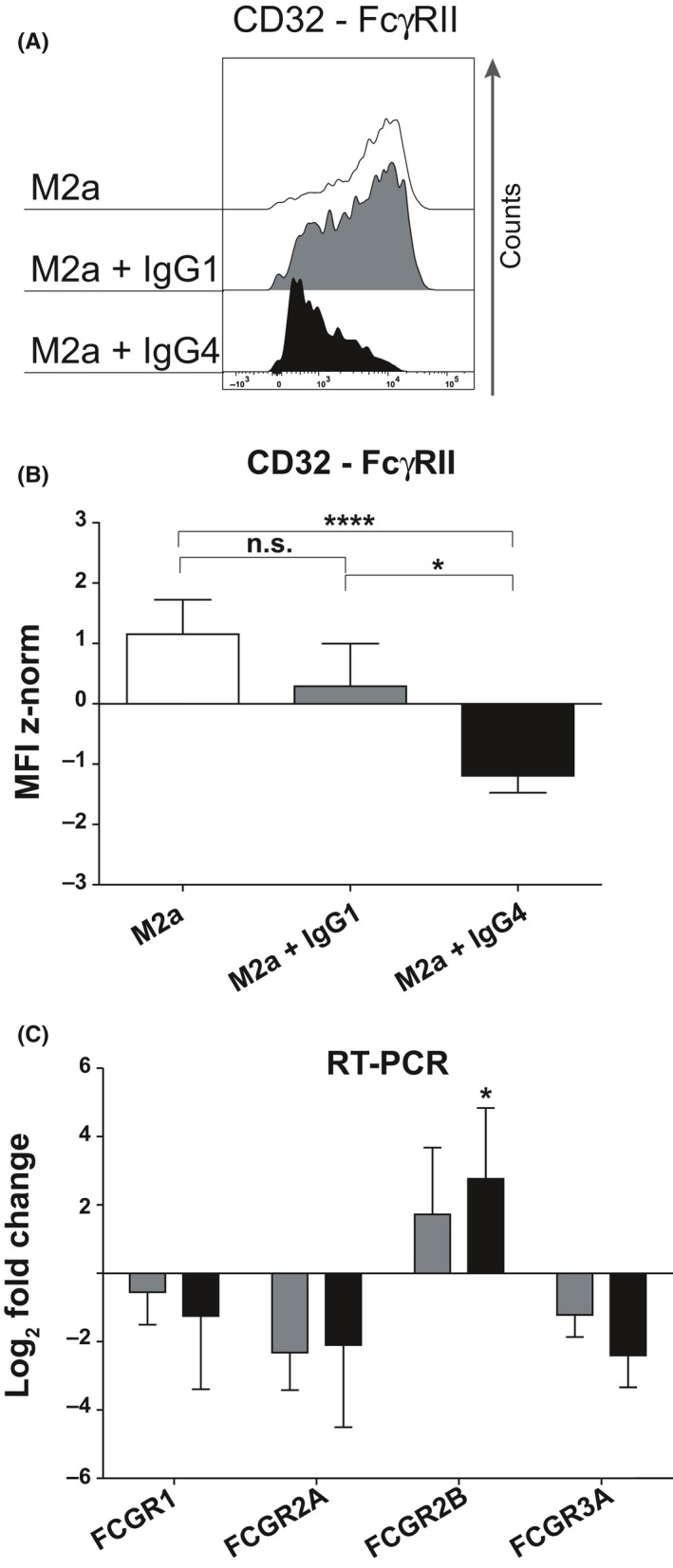
Analysis of FcγRII‐CD32 expressed by M2a macrophages polarized *in vitro* treated with myeloma (m)IgG1 or mIgG4 complexed on the plate. A, Flow cytometric analysis of cells polarized *in vitro* for 72 h. Half‐offset representation of the down‐modulation of FcγRII‐CD32 expression on M2a macrophages upon mIgG4 treatment in one representative individual (six donors. in three independent experiments); *y*‐axis: events normalized to the mode for FcγRII‐CD32; empty graphs: staining of M2a cells; gray: M2a incubated with complexed mIgG1 (M2a + IgG1); black: M2a + IgG4. B, MFI z‐normalization was performed for FcγRII‐CD32 for each donor before statistical validation. White bars: M2a macrophages; gray bars: M2a cells +IgG1; black bars: M2a cells +IgG4. The results from three independent experiments were combined for statistical analysis. Repeated‐measures one‐way ANOVA statistical analysis and Tukey multiple comparison post‐hoc test were performed, *P < 0.05, **P < 0.01, ***P < 0.001, ****P < 0.0001, n.s. not significant. C, Logarithmic 2‐fold change in FcγR mRNA obtained from M2a macrophages polarized *in vitro* for 48 h treated with mIgG1 or mIgG4. Repeated‐measures two‐way ANOVA statistical analysis and Tukey multiple comparison post‐hoc test were performed, *P < 0.05

Next, we examined which FcγRs could play a role in transferring an IgG subclass‐dependent signal to macrophages. M2a macrophages express almost exclusively FcγRII‐CD32 (Figure 1B), and this expression was not affected by ICs containing IgG1 compared to M2a macrophages only (Figure 5A,B); however, a statistically significant down modulation of the surface expression of FcγRII‐CD32 was seen in M2a when treated with complexed IgG4 compared to IgG1 (*P* = 0.0335), or without ICs (*P* < 0.0001). The different expressions of all the FcγRs expression are summarized in Figure S3B. Moreover, the treatment of M2a with IC‐complexed IgG1 or IC‐complexed IgG4 shown a down modulation FCGRIIA mRNA and an upregulation of FCGRIIB mRNA in comparison with M2a (ICs with mIgG4 *P* = 0.0259) (Figure 5C).

### 3.6 Allergen‐specific M2a modulation toward an M2b‐like regulatory phenotype

**Figure 6:**
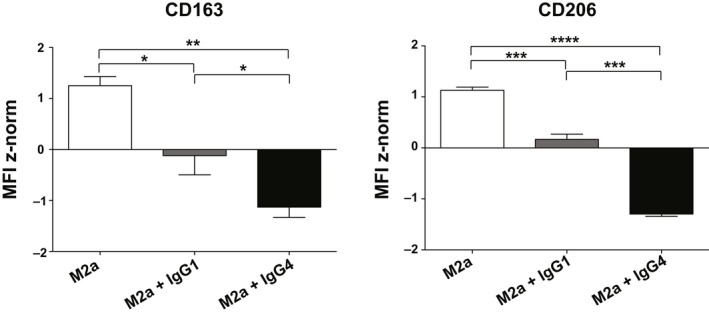
Flow cytometric analysis of surface markers expressed by M2a macrophages after 72 h *in vitro* polarization. The M2a macrophages polarized *in vitro* in the presence of anti‐Phl p 5 IgG1 or IgG4 antibodies binding to plate‐coated rPhl p 5. MFI z‐normalization was performed to normalize the MFI of each marker for each donor before statistical validation. White bars: M2a cells; gray bars: M2a cells +IgG1; black bars: M2a cells +IgG4. The results from three independent experiments were combined for statistical analysis. Repeated‐measures one‐way ANOVA statistical analysis and Tukey multiple comparison post‐hoc test were performed, *P < 0.05, **P < 0.01, ***P < 0.001, ****P < 0.0001

To translate the findings with myeloma IgG above into an allergen‐specific model, we applied human anti‐Phl p 5‐specific IgG1^20^, ^21^ and IgG4 antibodies. Only when M2a polarized macrophages were stimulated with αPhlp5‐IgG4 ICs, the surface expressions of CD163 (*P* = 0.0415) and CD206 (*P* = 0.0001) were significantly reduced, in comparison with the IgG1 isotype treatment (Figure 6).

## 4 DISCUSSION

Allergen‐specific immunotherapy (AIT) has been used for over 100 years as the only curative treatment for IgE‐mediated allergy that confers long‐term clinical benefit.^9^, ^10^ Regulatory T and B cells as sources of IL‐10 are thereby central in the induction of immune tolerance to the specific allergen.^10^ In fact, IL‐10 suppresses the allergic inflammatory response by limiting the production of pro‐inflammatory chemokines and cytokines and, in parallel, enhances the survival, proliferation, differentiation, and isotype switching of human B cells suppressing IgE and promoting the IgG4 subclass.^1^, ^10^, ^22^ IgG blocking antibodies can complex the allergen and then via FcγRIIb on effector cells dampen their IgE‐mediated anaphylactic reaction. Among all, the IgG4 subclass has an outstanding anti‐inflammatory potency. This has been explained by the fact that it cannot fix complement, and that it is the only class that may be naturally bispecific due to Fab arm exchange,^23^ limiting its capacity to be cross‐linked by an allergen.^24^ The non‐inflammatory action of IgG4 is desired in allergy, but harmful in other conditions with overshooting immune tolerance, like cancer.^25^, ^26^ The tremendous increase in IgG4 is a common characteristic of AIT. The current model is that IL‐10 from regulatory T and B cells decreases the (IL‐4‐induced) IgE production by B cells in favor of IL‐4‐induced IgG4 production.^1^, ^10^

We addressed here whether IgG4 might even have a more prominent role in immune tolerance by directly influencing IL‐10 production. Corresponding to the Th1‐Th2 paradigm, also macrophages can functionally differentiate into M1, and M2 cells, with the M2a, b, c subtypes, which can be mimicked by *in vitro* stimulation protocols.^16^, ^27^ It caught our attention that especially the tolerogenic M2b subtype is activated in the presence of IgG immune complexes, which so far were not further classified.

The study hypothesis was, therefore, that pro‐allergic M2a macrophages could by immune complexes containing IgG4, as induced by AIT, be functionally converted into an immunoregulatory M2b subtype. M2b‐derived secretion of CCL1 and IL‐10 may contribute to the development of a tolerogenic microenvironment.

We first determined the phenotype and the secretion pattern of all *in vitro* polarized macrophages because of some overlap in their characteristics. For instance, the expression of CD206 that is considered high in the M2a subtype is also expressed by M2o or M2c (Figure 1A) and even by M1 cells;^5^ CD163, which is medium‐low expressed by M2a and in M2o subtypes, is upregulated by glucocorticoid treatment in the M2c subtype.^4^, ^5^, ^16^ In accordance with previous studies, only the M2b macrophage subtype produced CCL1 chemokine and higher IL‐10 levels than any other subtypes and thus had the most pronounced signature.^12^, ^13^, ^14^

It is known that macrophages have a degree of plasticity, but the phenotypic and functional change in pro‐allergic M2a toward an M2b‐like phenotype only in the presence of IgG4, but not IgG1, was striking. This subclass‐specific phenomenon was observed with complexes containing myeloma IgG4, as well as when using recombinant anti‐Phl p 5 specific IgG4 when complexed to its specific allergen. Therefore, IgG4 complexes are potent stimulators of macrophages to CCL‐1 and IL‐10 production and capable to turn them from a pro‐allergic to a regulatory phenotype.

To investigate the molecular mechanism, we examined the surface expression of the FcγR family on M2a macrophages. Only M2o and M2c macrophages expressed high‐affinity FcγRI and low‐affinity FcγRIII (Figure 1B), both characterized by an immunoreceptor tyrosine‐based activation motif (ITAM) in the cytoplasmic portion. IgG1 has a higher binding affinity for FcγRI and FcγRIII than IgG4, which binds only to FcγRI with high affinity (K_a_ 3 × 10^7 ^M^−1^).^2^, ^3^, ^28^, ^29^ On the contrary, FcγRII is expressed by all macrophage subtypes, but in the IL‐4‐activated M2a subtype, the inhibitory FcγRIIb receptor subtype, bearing an immunoreceptor tyrosine‐based inhibition motif (ITIM), is higher expressed.^30^, ^31^ IgG4 is the only IgG subclass that binds both receptors, FcγRIIa and FcγRIIb, with the same affinity (both K_a_ 2 × 10^5 ^M^−1^) in comparison with IgG1 (K_a_ 4 × 10^6^ and K_a_ 1 × 10^5 ^M^−1^, respectively).^2^, ^3^, ^29^ Moreover, the co‐engagement of the FcγRIIb with any other activatory FcγRs resulted in an inhibitory response of the effector cells,^28^ as might happen also in M2a macrophage effector cells.

M2b macrophages released CCL1 upon IgG incubation which was correlated with FcγRII binding, without analysis of the specific IgG subclasses.^12^ Our data show that IgG4‐mediated FcγRII stimulation is decisive for the phenotypic and functional conversion of M2a into M2b‐like macrophages with subsequent CCL1 secretion. IgG4 binds both FcγRIIa and FcγRIIb, though in an allergic IL‐4‐rich environment, more inhibitory FcγRIIb are expressed on M2a macrophages, and available for IgG4 binding.^32^

High doses of allergens are needed in AIT to achieve immune tolerance,^28^ and both IgG1 and IgG4 are formed in different ratios. Only when enough IgG4 compared to IgG1 is produced, the FcγRIIb on M2a macrophages will be engaged and lead to a M2b conversion, resulting in secretion of IL‐10 and CCL1. While IL‐10 further supports the class switch of B cells in IgG4 producing cells, CCL1 will recall CCR8^+^FOXP3^+^ Tregs from the periphery.

Our results are in line with studies on IgG4‐related disease (IgG4‐RD), a fibro‐inflammatory condition characterized by abundant IgG4^+^ plasma cells in affected tissues, and by specific involvement of Th2, Treg cells, and M2 macrophages.^33^, ^34^, ^35^ A Japanese genomewide association study of IgG4‐RD reported three susceptibility loci consistent with an antigen‐driven disease. Currently, IgG4 is rather regarded as a protective antibody in IgG4‐RD, dampening the more harmful effects of IgG1.^36^ In accordance, FCGR2B is considered to play a critical role in the control of IgG4‐RD.^37^ A recent study correlates the high expression of IgG4 from IgG4‐RD sclerosing cholangitis patients’ B cells with the high expression of CCL1 in the tissues and, in turn, with the recruitment of CCR8^+^FOXP3^+^ Treg cells.^38^ The high affinity of CCL1 for CCR8 as well as the emerging opinion that CCL1 is indispensable for Treg function, further emphasizes the link of CCL1 and IL‐10 whit the creation of an immunoregulatory microenvironment.^15^, ^39^ Moreover, the importance of CCL1, produced by M2b macrophages, in this process is also documented by the fact that treatment with antisense CCL1‐oligonucleotide inhibited M2b macrophages to maintain their characteristics such as IL‐10 production and inflammation inhibition.^13^, ^14^

Thus, our data argue for a central role of IgG4 in immune tolerance as induced by AIT, prompting cross talk between macrophages and regulatory lymphocytes.

## CONFLICTS OF INTEREST

The authors declare that they have no conflicts of interest.

## AUTHOR CONTRIBUTIONS

EJJ and RB designed the study; RB, EJJ, and FRW conceived and designed the experiments; RB and AOR performed the experiments; MBF, KH, AOR, and SF contributed to reagents and materials; RB, AOR, and EJJ analyzed the data; RB, EJJ, and HS contributed to the interpretation of the data; RB and EJJ drafted the paper. All authors contributed to texts and approved the manuscript.

## References

[1] Fujita H , Soyka MB , Akdis M , Akdis CA . Mechanisms of allergen‐specific immunotherapy. Clin Transl Allergy. 2012;2:2. 22409879 10.1186/2045-7022-2-2 PMC3395833

[2] Bruhns P , Iannascoli B , England P , et al. Specificity and affinity of human Fcgamma receptors and their polymorphic variants for human IgG subclasses. Blood. 2009;113:3716‐3725. 19018092 10.1182/blood-2008-09-179754

[3] Guilliams M , Bruhns P , Saeys Y , Hammad H , Lambrecht BN . The function of Fcgamma receptors in dendritic cells and macrophages. Nat Rev Immunol. 2014;14:94‐108. 24445665 10.1038/nri3582

[4] Roszer T . Understanding the Mysterious M2 macrophage through activation markers and effector mechanisms. Mediators Inflamm. 2015;2015:816460. 26089604 10.1155/2015/816460 PMC4452191

[5] Porcheray F , Viaud S , Rimaniol AC , et al. Macrophage activation switching: an asset for the resolution of inflammation. Clin Exp Immunol. 2005;142:481‐489. 16297160 10.1111/j.1365-2249.2005.02934.x PMC1809537

[6] Deszo EL , Brake DK , Kelley KW , Freund GG . IL‐4‐dependent CD86 expression requires JAK/STAT6 activation and is negatively regulated by PKCdelta. Cell Signal. 2004;16:271‐280. 14636897 10.1016/s0898-6568(03)00137-2

[7] Mantovani A , Sica A , Sozzani S , Allavena P , Vecchi A , Locati M . The chemokine system in diverse forms of macrophage activation and polarization. Trends Immunol. 2004;25:677‐686. 15530839 10.1016/j.it.2004.09.015

[8] Gordon S . Alternative activation of macrophages. Nat Rev Immunol. 2003;3:23‐35. 12511873 10.1038/nri978

[9] Akdis CA , Akdis M . Mechanisms of allergen‐specific immunotherapy and immune tolerance to allergens. World Allergy Organ J. 2015;8:17. 26023323 10.1186/s40413-015-0063-2 PMC4430874

[10] Shamji MH , Kappen JH , Akdis M , et al. Biomarkers for monitoring clinical efficacy of allergen immunotherapy for allergic rhinoconjunctivitis and allergic asthma: an EAACI Position Paper. Allergy. 2017;72:1156‐1173. 28152201 10.1111/all.13138

[11] Meiler F , Klunker S , Zimmermann M , Akdis CA , Akdis M . Distinct regulation of IgE, IgG4 and IgA by T regulatory cells and toll‐like receptors. Allergy. 2008;63:1455‐1463. 18925882 10.1111/j.1398-9995.2008.01774.x

[12] Sironi M , Martinez FO , D'Ambrosio D , et al. Differential regulation of chemokine production by Fcgamma receptor engagement in human monocytes: association of CCL1 with a distinct form of M2 monocyte activation (M2b, Type 2). J Leukoc Biol. 2006;80:342‐349. 16735693 10.1189/jlb.1005586

[13] Asai A , Nakamura K , Kobayashi M , Herndon DN , Suzuki F . CCL1 released from M2b macrophages is essentially required for the maintenance of their properties. J Leukoc Biol. 2012;92:859‐867. 22730547 10.1189/jlb.0212107

[14] Asai A , Tsuchimoto Y , Ohama H , et al. Host antitumor resistance improved by the macrophage polarization in a chimera model of patients with HCC. Oncoimmunology. 2017;6:e1299301. 28507807 10.1080/2162402X.2017.1299301 PMC5414886

[15] Barsheshet Y , Wildbaum G , Levy E , et al. CCR8 + FOXp3+ Treg cells as master drivers of immune regulation. Proc Natl Acad Sci USA. 2017;114:6086‐6091. 28533380 10.1073/pnas.1621280114 PMC5468670

[16] Martinez FO , Gordon S . The M1 and M2 paradigm of macrophage activation: time for reassessment. F1000Prime Rep. 2014;6:13. 24669294 10.12703/P6-13 PMC3944738

[17] Swisher JF , Haddad DA , McGrath AG , Boekhoudt GH , Feldman GM . IgG4 can induce an M2‐like phenotype in human monocyte‐derived macrophages through FcgammaRI. mAbs. 2014;6:1377‐1384. 25484046 10.4161/19420862.2014.975657 PMC4622582

[18] Genovese A , Borgia G , Bjorck L , et al. Immunoglobulin superantigen protein L induces IL‐4 and IL‐13 secretion from human Fc epsilon RI+ cells through interaction with the kappa light chains of IgE. J Immunol. 2003;170:1854‐1861. 12574351 10.4049/jimmunol.170.4.1854

[19] Ye J , Coulouris G , Zaretskaya I , Cutcutache I , Rozen S , Madden TL . Primer‐BLAST: a tool to design target‐specific primers for polymerase chain reaction. BMC Bioinformatics. 2012;13:134. 22708584 10.1186/1471-2105-13-134 PMC3412702

[20] Flicker S , Vrtala S , Steinberger P , et al. A human monoclonal IgE antibody defines a highly allergenic fragment of the major timothy grass pollen allergen, Phl p 5: molecular, immunological, and structural characterization of the epitope‐containing domain. J Immunol. 2000;165:3849‐3859. 11034391 10.4049/jimmunol.165.7.3849

[21] Madritsch C , Flicker S , Scheiblhofer S , et al. Recombinant monoclonal human immunoglobulin E to investigate the allergenic activity of major grass pollen allergen Phl p 5. Clin Exp Allergy. 2011;41:270‐280. 21143538 10.1111/j.1365-2222.2010.03666.x

[22] Bohm L , Maxeiner J , Meyer‐Martin H , et al. IL‐10 and regulatory T cells cooperate in allergen‐specific immunotherapy to ameliorate allergic asthma. J Immunol. 2015;194:887‐897. 25527785 10.4049/jimmunol.1401612

[23] Jensen‐Jarolim E , Turner MC , Karagiannis SN . AllergoOncology: IgE‐ and IgG4‐mediated immune mechanisms linking allergy with cancer and their translational implications. J Allergy Clin Immunol. 2017;140:982‐984. 28526623 10.1016/j.jaci.2017.04.034

[24] Davies AM , Sutton BJ . Human IgG4: a structural perspective. Immunol Rev. 2015;268:139‐159. 26497518 10.1111/imr.12349 PMC4670484

[25] Jensen‐Jarolim E , Bax HJ , Bianchini R , et al. AllergoOncology: opposite outcomes of immune tolerance in allergy and cancer. Allergy. 2018;73:328‐340. 28921585 10.1111/all.13311 PMC6038916

[26] Crescioli S , Correa I , Karagiannis P , et al. IgG4 characteristics and functions in cancer immunity. Curr Allergy Asthma Rep. 2016;16:7. 26742760 10.1007/s11882-015-0580-7 PMC4705142

[27] Sica A , Mantovani A . Macrophage plasticity and polarization: *in vivo* veritas. J Clin Invest. 2012;122:787‐795. 22378047 10.1172/JCI59643 PMC3287223

[28] James LK , Till SJ . Potential mechanisms for IgG4 inhibition of immediate hypersensitivity reactions. Curr Allergy Asthma Rep. 2016;16:23. 26892721 10.1007/s11882-016-0600-2 PMC4759210

[29] Bruhns P , Jonsson F . Mouse and human FcR effector functions. Immunol Rev. 2015;268:25‐51. 26497511 10.1111/imr.12350

[30] Ambarus CA , Santegoets KC , van Bon L , et al. Soluble immune complexes shift the TLR‐induced cytokine production of distinct polarized human macrophage subsets towards IL‐10. PLoS One. 2012;7:e35994. 22563430 10.1371/journal.pone.0035994 PMC3338562

[31] Anthony RM , Kobayashi T , Wermeling F , Ravetch JV . Intravenous gammaglobulin suppresses inflammation through a novel T(H)2 pathway. Nature. 2011;475(7354):110‐113. 21685887 10.1038/nature10134 PMC3694429

[32] Ambarus CA , Krausz S , van Eijk M , et al. Systematic validation of specific phenotypic markers for *in vitro* polarized human macrophages. J Immunol Methods. 2012;375(1–2):196‐206. 22075274 10.1016/j.jim.2011.10.013

[33] Della‐Torre E , Lanzillotta M , Doglioni C . Immunology of IgG4‐related disease. Clin Exp Immunol. 2015;181:191‐206. 25865251 10.1111/cei.12641 PMC4516435

[34] Furukawa S , Moriyama M , Tanaka A , et al. Preferential M2 macrophages contribute to fibrosis in IgG4‐related dacryoadenitis and sialoadenitis, so‐called Mikulicz's disease. Clin Immunol. 2015;156:9‐18. 25450336 10.1016/j.clim.2014.10.008

[35] Wolfson AR , Hamilos DL . Recent advances in understanding and managing IgG4‐related disease. F1000Res. 2017;23:6. pii: F1000. 10.12688/f1000research.9399.1 PMC5325071 28299186

[36] Trampert DC , Hubers LM , van de Graaf SFJ , Beuers U . On the role of IgG4 in inflammatory conditions: lessons for IgG4‐related disease. Biochim Biophys Acta. 2018;1864(4 Pt B):1401‐1409. 10.1016/j.bbadis.2017.07.038 28782655

[37] Haldar D , Hirschfield GM . Deciphering the biology of IgG4‐related disease: specific antigens and disease? Gut. 2018;67:602‐605. 29101259 10.1136/gutjnl-2017-314861 PMC6058063

[38] Zen Y , Liberal R , Nakanuma Y , Heaton N , Portmann B . Possible involvement of CCL1‐CCR8 interaction in lymphocytic recruitment in IgG4‐related sclerosing cholangitis. J Hepatol. 2013;59:1059‐1064. 23811304 10.1016/j.jhep.2013.06.016

[39] Hoelzinger DB , Smith SE , Mirza N , Dominguez AL , Manrique SZ , Lustgarten J . Blockade of CCL1 inhibits T regulatory cell suppressive function enhancing tumor immunity without affecting T effector responses. J Immunol. 2010;184:6833‐6842. 20483762 10.4049/jimmunol.0904084

