# Supplementary figures and images for: IgG4 drives M2a macrophages to a regulatory M2b‐like phenotype: potential implication in immune tolerance
Source: Allergy. 2018 Nov 28;74(3):483–94. doi: 10.1111/all.13635 (PMC6492166; doi:10.1111/all.13635)

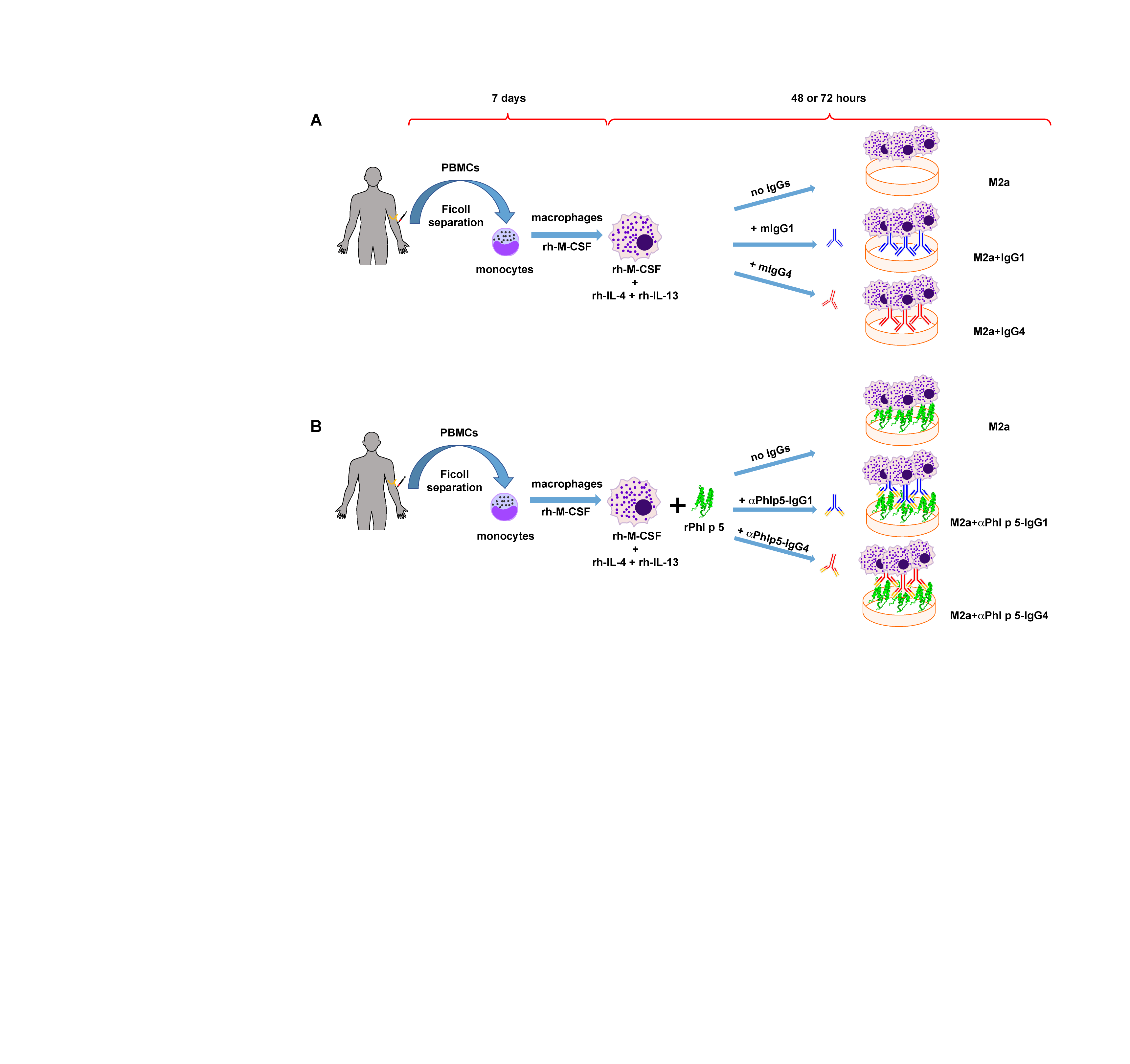

Supplement: Supplementary file 1 [file ALL-74-483-s001.tif]

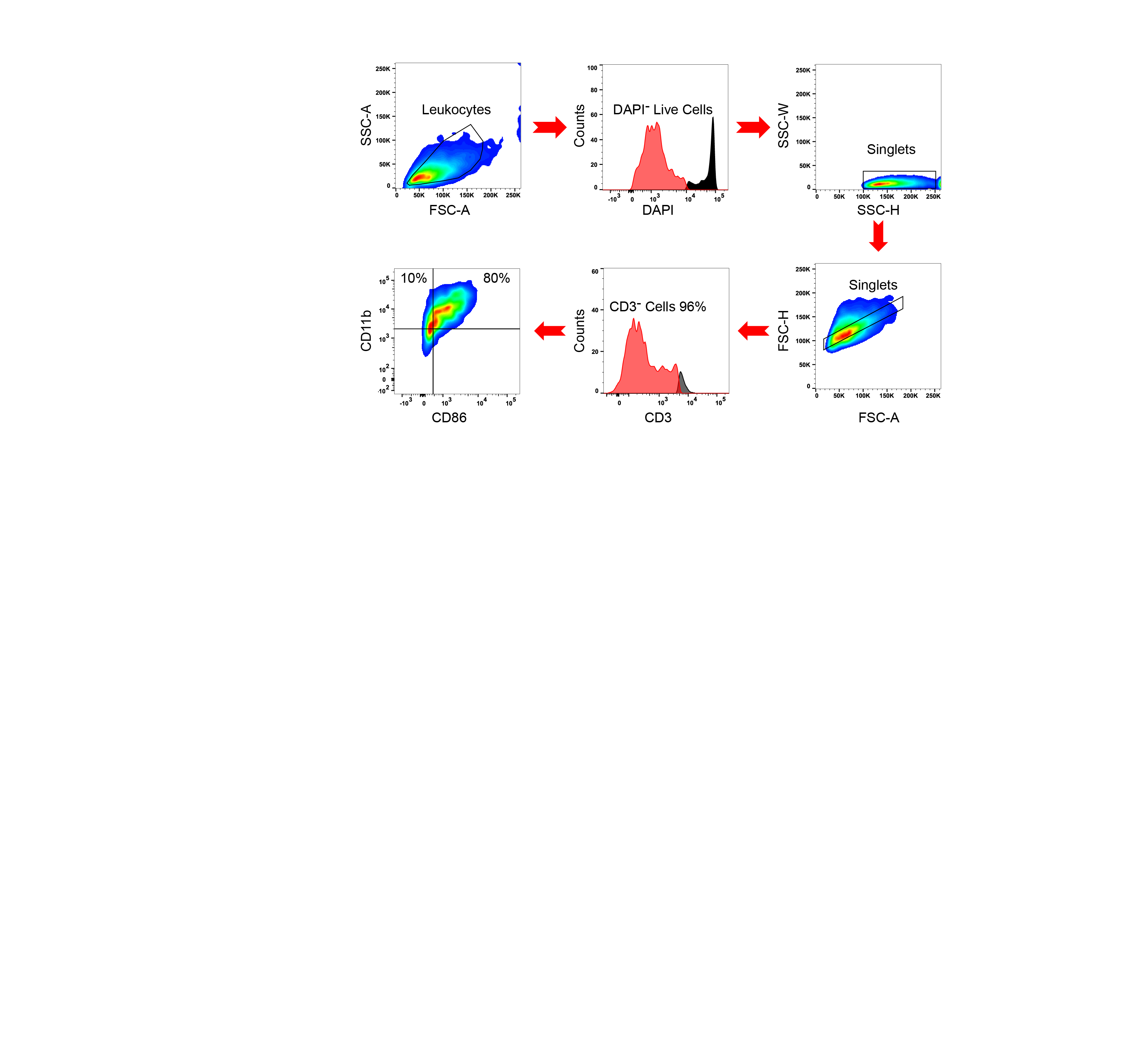

Supplement: Supplementary file 2 [file ALL-74-483-s002.tif]

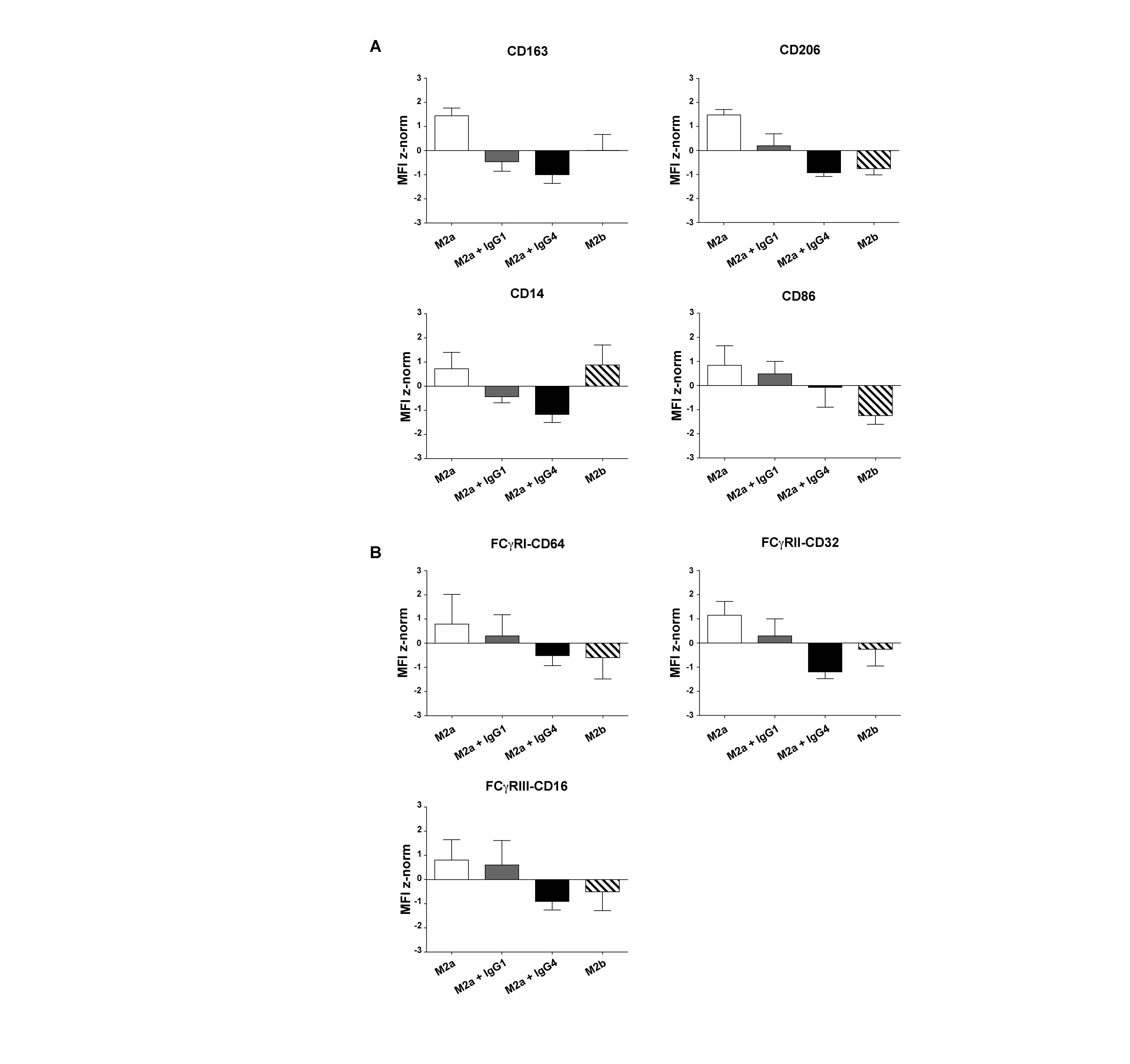

Supplement: Supplementary file 3 [file ALL-74-483-s003.tif]

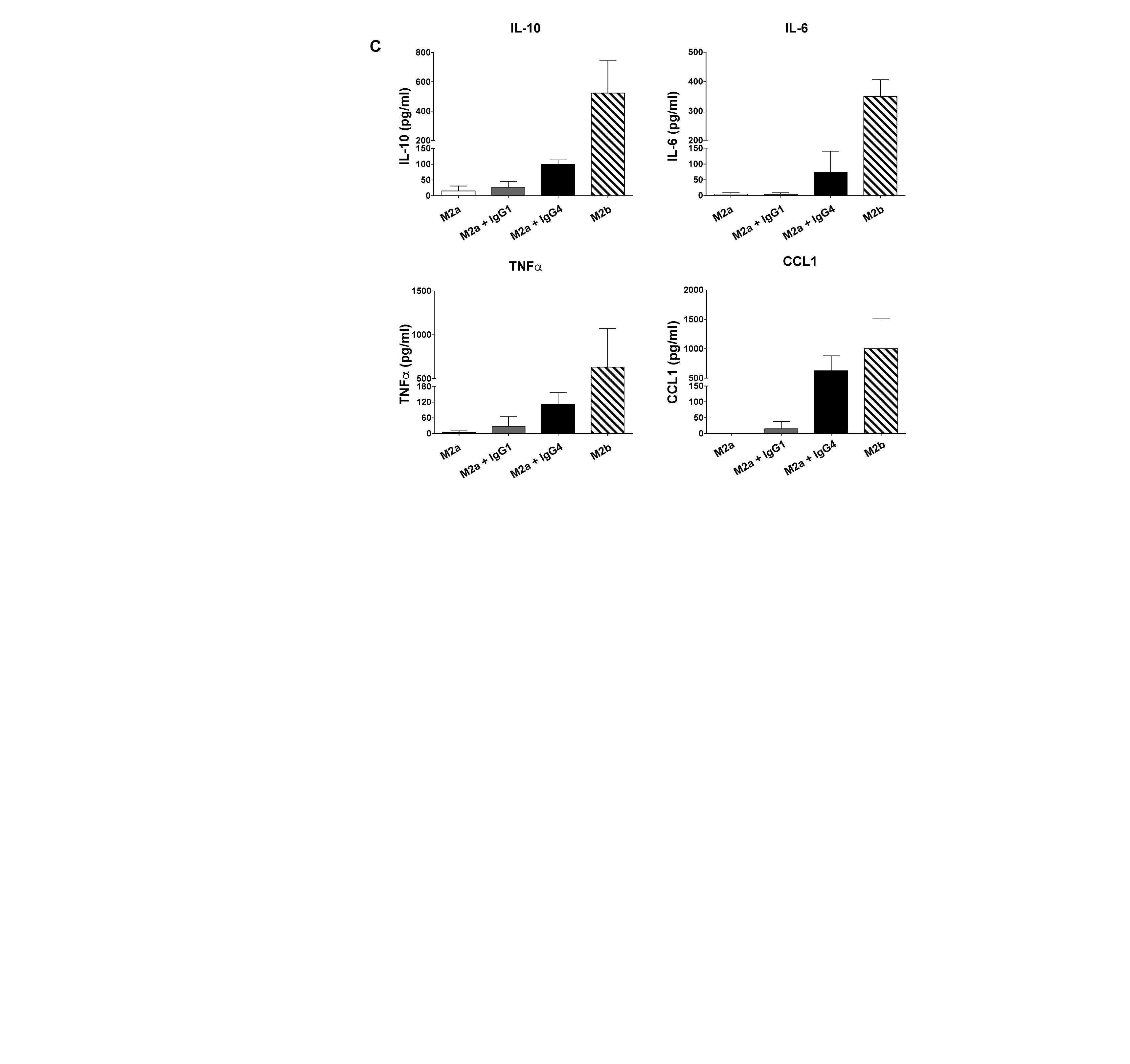

Supplement: Supplementary file 4 [file ALL-74-483-s004.tif]
