# Supplementary material for: IgG4 drives M2a macrophages to a regulatory M2b‐like phenotype: potential implication in immune tolerance
Source: Allergy. 2018 Nov 28;74(3):483–94. doi: 10.1111/all.13635 (PMC6492166; doi:10.1111/all.13635)
Supplement: Supplementary file 5 [file ALL-74-483-s005.docx]

**Supporting information**

**IgG4 drives M2a macrophages to a regulatory M2b-like phenotype: potential implication in immune tolerance.**

**Running title:** IgG4 reshape M2a in regulatory M2b-like macrophages

Rodolfo Bianchini^1^, Franziska Roth-Walter^1^, Anna Ohradanova-Repic^2^, Sabine Flicker^3^, Karin Hufnagl^1^, Michael Bernhard Fischer^4,5^, Hannes Stockinger^3^ and Erika Jensen-Jarolim*^1,3^

1. The Interuniversity Messerli Research Institute, University of Veterinary Medicine Vienna, Medical University of Vienna, Dept. of Comparative Medicine (Vienna, Austria);
2. Institute for Hygiene and Applied Immunology, Center for Pathophysiology, Infectiology and Immunology, Medical University of Vienna (Vienna, Austria);
3. Institute of Pathophysiology and Allergy Research, Center for Pathophysiology, Infectiology and Immunology, Medical University of Vienna (Vienna, Austria);
4. Medical University Vienna, Dept. of Blood Group Serology and Transfusion Medicine (Vienna, Austria);
5. Danube University Krems, Dept. of Health Science and Biomedicine, (Vienna, Austria).

**Protocol for isolation and treatment of human monocytes**

Peripheral blood from nine volunteers were obtained by venipuncture and evacuated tube system of collection for fresh blood in 9 ml vacuum tube coated with lithium heparin (Greiner Bio-One, Austria) or from LRS and a TrimaAccel automated blood collection system (Terumo BCT, Lakewood, CA, USA), and diluted 1:4 in Hank's Balanced Salt Solution (HBSS) (GE Healthcare, Solingen, Germany). Peripheral blood mononuclear cells (PBMCs) were isolated by Ficoll-Paque (GE Healthcare, Solingen, Germany) (density 1.077 g/ml) gradient centrifugation (400×g, 30 min, 20 °C, in a swinging-bucket rotor without brake). The mononuclear white layer was recovered, resuspended in RPMI 1640, and cells seeded in 6 well plates (Falcon, Corning, NY), 2x10^6^ cells/ml, 3.5 ml / well. After 2 hours, non-adherent cells were washed away 2 times and adherent cells (monocytes) were maintained in RPMI 1640 with 10% heat-inactivated FBS, and 1% of P/S (cRPMI), supplemented with 20ng/ml rh-M-CSF for 7-9 days. Half of the medium was refreshed every 2-3 days. The purity of adherent cells after 3 days was analysed by a multicolor staining mix of primary monoclonal antibodies against CD3, CD86 and CD11b or isotype controls (described in Table S2 A) diluted 1:100 for each specific antibody. Dead cells were excluded by dead cell staining performed with SYTOX™ Green Ready Flow™ Reagent (Thermo Fisher Scientific, Waltham, MA, USA).

**Protocols for immune complex formation with IgG1 and IgG4:**

To produce plate-bound immune complex of human myeloma IgG1 (mIgG1) or IgG4 (mIgG4), a 96 well plate (Falcon, Corning, NY) was coated with mIgG1 or mIgG4 antibodies, 5 μg/well in HBSS, at 37°C, 5% CO_2_, 95% humidity for 1h, and then washed twice with 200 μl of cRPMI.

To produce immune complexes of human myeloma IgG1 (mIgG1) or IgG4 (mIgG4) in solution, 7-9 days differentiated monocytes (see above) were incubated at room temperature for 30 minutes with either mIgG1 or mIgG4 antibodies at a concentration of 0.35µM in HBSS, and then washed twice with 200 μl of cRPMI. The cells were subsequently treated with rProtein-L (Thermo Fisher Scientific, Waltham, MA) at a ratio of 1:4 (Protein L : myeloma IgG1 or IgG4 antibodies).

To produce immune complexes of human anti-Phl p 5 IgG1 (αPhl p 5-IgG1) or IgG4 (αPhl p 5-IgG4) with Phl p 5 allergen, a 96 well plate (Falcon, Corning, NY) was coated with 2 μg/well r-Phl p 5 (Biomay, Vienna, Austria, endotoxin content 0.003EU/μg) in 100 mM sodium carbonate buffer, pH 9.5, at 37°C, 5% CO_2_, 95% humidity for 1 h, and then washed twice with 200 μl of cRPMI.

Then wells were saturated with T-HBSS/3% BSA, for 1h at 37°C, 5% CO_2_, 95% humidity, and washed with HBSS. Subsequently, 5 μg/well αPhl p 5-IgG1 or αPhl p 5-IgG4 antibodies in HBSS were added, incubated for 1 hour, and unbound mAbs were washed away with T-HBSS and once with cRPMI.

**Protocol for surface markers staining:**

After 72 hours, cells were collected by treatment with ice-cold PBS/EDTA, twice for 15 min on ice. Cells were then washed with staining buffer (HBSS supplemented with 3% FBS) at 4°C. A multicolor staining mix was prepared with antibodies diluted 1:100 for each specific surface marker antibody (described in Table S2 A). Similarly, a multicolor staining mix was prepared consisting of isotype controls for each antibody (described in Table S2). The cells were stained at 4°C for 30 min, then washed once with FACS buffer and resuspended in 250 μl FACS buffer for acquisition.

**Protocol for FcγRs staining:**

For the FcγRs staining, the cells were detached as described above using ice-cold PBS^-Ca-Mg^/EDTA, washed with an ice-cold staining buffer (PBS^-Ca-Mg^ plus 1% BSA, 0.02% NaN_3_) and then blocked with 2.4 mg/ml human IgG (Beriglobin P; CSL Behring, King of Prussia, PA) in staining buffer for 30 min on ice. The cells were then incubated with a multicolor staining mix of primary monoclonal antibodies against CD64, CD32 and CD16 or isotype controls (as specified in Table S2 B; diluted 1:40 to 1:80 with the staining buffer) at 4°C for 30 min and washed twice with the staining buffer. Prior analysis, 0.5 μg/ml 4',6-diamidino-2-phenylindole (DAPI) was added to cells and samples were acquired using an LRSII flow cytometer (Becton Dickinson, Franklin Lakes, NJ). Data were analyzed with the FlowJo software version 10.3 (FlowJo, LLC, Ashland, OR) and dead cells were excluded from the analysis based on DAPI fluorescence.

**Calculation of MFI z normalization:**

For the normalization of the geometric mean of fluorescence intensity (MFI z-norm) was used the following formula:

$$MFI znorm= \frac{\left( x-\bar{x} \right)}{S}$$

where $\bar{x}$ is the mean of the MFI value of the population and $S$ is the standard deviation of the MFI value of the population.

**Table S1:** Description of reagents used in this study.

| **Reagents** | **Acronyms** | **Provider** |
| --- | --- | --- |
| HyClone Hank’s Balanced Salt Solution 1x with calcium, magnesium, without phenol red | HBSS | GE Healthcare Life Sciences, South  Logan, UT |
| HyClone Dulbecco's Phosphate Buffered Saline without calcium and magnesium | PBS^-Ca-Mg^ | GE Healthcare Life Sciences, South  Logan, UT |
| Bovine serum albumin | BSA | Sigma-Aldrich Saint Louis, MO |
| Lipopolysaccharides from Escherichia coli O55:B5 (3x10^6^ EU/mg/ml) | LPS | Sigma-Aldrich Saint Louis, MO |
| Dexamethasone | Dex | Sigma-Aldrich Saint Louis, MO |
| Serum-free Roswell Park Memorial Institute 1640 medium with L-glutamine and sodium bicarbonate | RPMI | Sigma-Aldrich Saint Louis, MO |
| Fetal bovine sera | FBS | Thermo Fisher Scientific, Waltham, MA |
| Penicillin-Streptomycin antibiotic solution X100 | P/S | Sigma-Aldrich Saint Louis, MO |
| Ficoll-Paque Plus | Ficoll | GE Healthcare Life Sciences, South  Logan, UT |
| Tween-20 |  | Sigma-Aldrich Saint Louis, MO |
| Ethylenediaminetetraacetic acid disodium salt dehydrate | EDTA | Sigma-Aldrich Saint Louis, MO |
| Sodium bicarbonate |  | Merk, Kenilworth, NJ |
| Sodium carbonate |  | VWR, Vienna, Austria |
| SYTOX™ Green Ready Flow™ Reagent |  | Thermo Fisher Scientific, Waltham, MA |
| Recombinant human M-CSF | rh-M-CSF | BioLegend, San Diego, CA |
| Recombinant human IL-4 | rh-IL-4 | ImmunoTools, Friesoythe, Germany |
| Recombinant human IL-13 | rh-IL-13 | ImmunoTools, Friesoythe, Germany |
| Recombinant timothy grass pollen allergen Phl p 5 | rPhlp5 | Biomay, Vienna, Austria |
| Pierce™ Recombinant Protein L | rProtein-L | Thermo Fisher Scientific, Waltham, MA |
| Human myeloma-derived IgG1 | mIgG1 | Athens Research and Technology, Athens, GA |
| Human myeloma-derived IgG4 | mIgG4 | Athens Research and Technology, Athens, GA |
| Human anti-Phl p 5 mAb IgG1 | αPhlp5-IgG1 | Kindly provided by Sabine Flicker^1,2^ |
| Human anti-Phl p 5 mAb IgG4 | αPhlp5-IgG4 | Kindly provided by Sabine Flicker^1,2^ |

**Table S2 A:** Description of the antibodies used for surface marker staining analysis in this study.

| **Antibody** | **Clone** | **Fluorochrome** | **Provider** |
| --- | --- | --- | --- |
| anti-human mAb CD14 | 61D3 | FITC-labeled | Thermo Fisher Scientific, Waltham, MA |
| anti-human mAb CD11b | ICRF44 | PE/CY7-labeled | BioLegend, San Diego, CA |
| anti-human mAb CD86 | IT2.2 | PE-labeled | BioLegend, San Diego, CA |
| anti-human mAb CD163 | GHI/61 | APC-labeled | BioLegend, San Diego, CA |
| anti-human mAb CD206 | 15-2 | APC/CY7-labeled | BioLegend, San Diego, CA |
| anti-human mAb CD3 | SK7 | APC/CY7-labeled | BioLegend, San Diego, CA |
| mouse IgG1 isotype control mAb | MOPC-21 | FITC-labeled | BioLegend, San Diego, CA |
| mouse IgG1 isotype control mAb | MOPC-21 | PE/CY7-labeled | BioLegend, San Diego, CA |
| mouse IgG2b isotype control mAb | MPC-11 | PE-labeled | BioLegend, San Diego, CA |
| mouse IgG1 isotype control mAb | MOPC-21 | APC-labeled | BioLegend, San Diego, CA |
| mouse IgG1 isotype control mAb | MOPC-21 | APC/CY7-labeled | BioLegend, San Diego, CA |

**Table S2 B:** Description of the antibodies used for FcγRs staining analysis in this study.

| **Antibody** | **Clone** | **Fluorochrome** | **Provider** |
| --- | --- | --- | --- |
| anti-human mAb FcγRI-CD64 | 10.1 | FITC-labeled | EXBIO Praha, Vestec, Czech Republic |
| anti-human mAb FcγRII-CD32 | IV.3 | Pacific Blue-labeled (in house) |  |
| anti-human mAb FcγRII-CD32 | FUN2 | PE-labeled | BioLegend, San Diego, CA |
| anti-human mAb FcγRIII-CD16 | 3G8 | PerCP/Cy5.5-labeled | BioLegend, San Diego, CA |
| mouse IgG1 isotype control mAb | MOPC-21 | FITC-labeled | BioLegend, San Diego, CA |
| mouse IgG1 isotype control mAb | MOPC-21 | PerCP/Cy5.5-labeled | BioLegend, San Diego, CA |
| mouse IgG2b isotype control mAb | PPV-02 | Pacific Blue-labeled (in house) | a kind gift of Prof. V. Horejsi, Academy of Sciences of the Czech Republic, Prague,  Czech Republic) |
| mouse IgG2b isotype control mAb | MPC-11 | PE-labeled | BioLegend, San Diego, CA |

**Table S3:** Detailed scheme of polarization

| **Macrophage subtype** | **rh-M-CSF** | **rh-IL-4** | **rh-IL-13** | **LPS**  ***E. coli* O55:B5** | **Immuno complex** | **Dex** |
| --- | --- | --- | --- | --- | --- | --- |
| M2o | 20ng/ml |  |  |  |  |  |
| M2a | 20ng/ml | 20ng/ml | 20ng/ml |  |  |  |
| M2b | 20ng/ml |  |  | 20ng/ml (60 EU) | 50µg/ml |  |
| M2c | 20ng/ml |  |  |  |  | 50 nM |

**Table S4 A:** Description of primer pairs used in this study.

| **Gene** | **Forward Primer** | **Tm (°C)** | **GC %** | **length** |
| --- | --- | --- | --- | --- |
| CD64 (FCGR1A) | GGCACCTACCATTGCTCAGGC | 63 | 62 | 21 |
| CD32a (FCGR2A) | GCTGCCCAATTTGAGCCACCT | 61 | 57 | 21 |
| CD32b (FCGR2B) | CCAGCTCTTCACCGATGGGG | 63 | 65 | 20 |
| CD16 (FCGR3A) | GCAGGGGGCTTTTTGGGAGT | 61 | 60 | 20 |
| ACTB | ATTGCCGACAGGATGCAGAA | 57 | 50 | 20 |
| **Gene** | **Reverse Primer** | **Tm (°C)** | **GC %** | **length** |
| CD64 (FCGR1A) | CCCTCCAGGAGTGGGGATGT | 63 | 65 | 20 |
| CD32a (FCGR2A) | TGGTCGTTGGGAGGAAGAGTCA | 62 | 55 | 22 |
| CD32b (FCGR2B) | GCTGGTTTCTCAGGGAGGGTCT | 64 | 60 | 22 |
| CD16 (FCGR3A) | CCTGTGTCCACTGCAAAAAGGAGT | 62 | 50 | 24 |
| ACTB | GCTGATCCACATCTGCTGGAA | 60 | 52 | 21 |

**Table S4 B:** Protocol for Real-time polymerase chain reaction

| **Stage** | **Cycles** | **Step** | **Temp.** | **Time** |
| --- | --- | --- | --- | --- |
| Hold stage | 1 | Initial Denaturation | 95 °C | 12 min |
| PCR stage | 45 | Denaturing | 95 °C | 15 secs |
|  |  | Primer Annealing | 57 °C | 20 secs |
|  |  | Extending | 72 °C | 20 secs |
| Melt Curve stage | 1 | Denaturing | 95 °C | 15 secs |
|  |  | Re-annealing | 60 °C | 1 min |
|  |  | Dissociation | 95 °C | 15 secs |

**Table S5 A:** Macrophage surface expression of different markers used to discriminate among the different subtype of polarized macrophages. The values represent the geometric mean of fluorescence intensity (MFI) ± STDEV.

| **Macrophage subpopulations** | **CD163** | **CD206** | **CD86** | **CD14** |
| --- | --- | --- | --- | --- |
| M2o | 1368  ± 1200 | 2735  ± 1996 | 1098  ± 238 | 945  ± 700 |
| M2a | 846  ± 163 | 5203  ± 1544 | 3285  ± 847 | 571  ± 83 |
| M2b | 816  ± 198 | 1758  ± 302 | 968  ± 30 | 781  ± 277 |
| M2c | 4900  ± 3200 | 8998  ± 1969 | 1460  ± 180 | 1303  ± 257 |

**Table S5 B:** Macrophage surface expression of different FcγRs among the different macrophage subtypes. The values represent the geometric mean of fluorescence intensity (MFI) ± STDEV.

| **Macrophage subpopulations** | **FcγRI-CD64** | **FcγRII-CD32** | **FcγRIII-CD16** |
| --- | --- | --- | --- |
| M2o | 276  ± 143 | 3818  ± 2510 | 148  ± 120 |
| M2a | 89.5  ± 60 | 4265  ± 2800 | 127  ± 130 |
| M2b | 0  ± 20 | 1173  ± 1819 | 15  ± 20 |
| M2c | 140  ± 95 | 7140  ± 4520 | 97.5  ± 88.5 |

**Table S6:** Overview of cytokine or chemokine expression levels of ~~by~~ M2a macrophages alone or after treatments with ICs consisting of mIgG1 or mIgG4 (mean of concentration ± STDEV). Statistical analysis was performed with repeated measures one-way ANOVA followed by Tukey multiple comparison post-hoc test. Level of significance are reported as p-values.

| **Cytokine/ Chemokine** | **M2a**  (pg/ml) | **M2a+IgG1** (pg/ml) | **M2a+IgG4**  (pg/ml) | **M2a** vs. **M2a+IgG1^1^** | **M2a** vs. **M2a+IgG4^1^** | **M2a+IgG1**vs. **M2a+IgG4^1^** |
| --- | --- | --- | --- | --- | --- | --- |
| IL-10 | 15.3 ± 15.6 | 27.5 ± 17.7 | 98.70 ± 14.6 | p=0.0170 | p< 0.0001 | p=0.0006 |
| IL-6 | 4.7 ± 4.3 | 4.2 ± 4.8 | 74.34 ± 66.1 | p=0.9443 | p=0.1223 | p=0.1192 |
| TNFα | 3.8 ± 6.1 | 27.8 ± 36.5 | 111.3 ± 45.0 | p=0.2492 | p=0.0051 | p=0.0394 |
| CCL1 | 0 | 15.1 ± 23.4 | 626.5 ± 252.7 | p=0.3350 | p=0.0041 | p=0.0051 |

***^1^*** p-values.


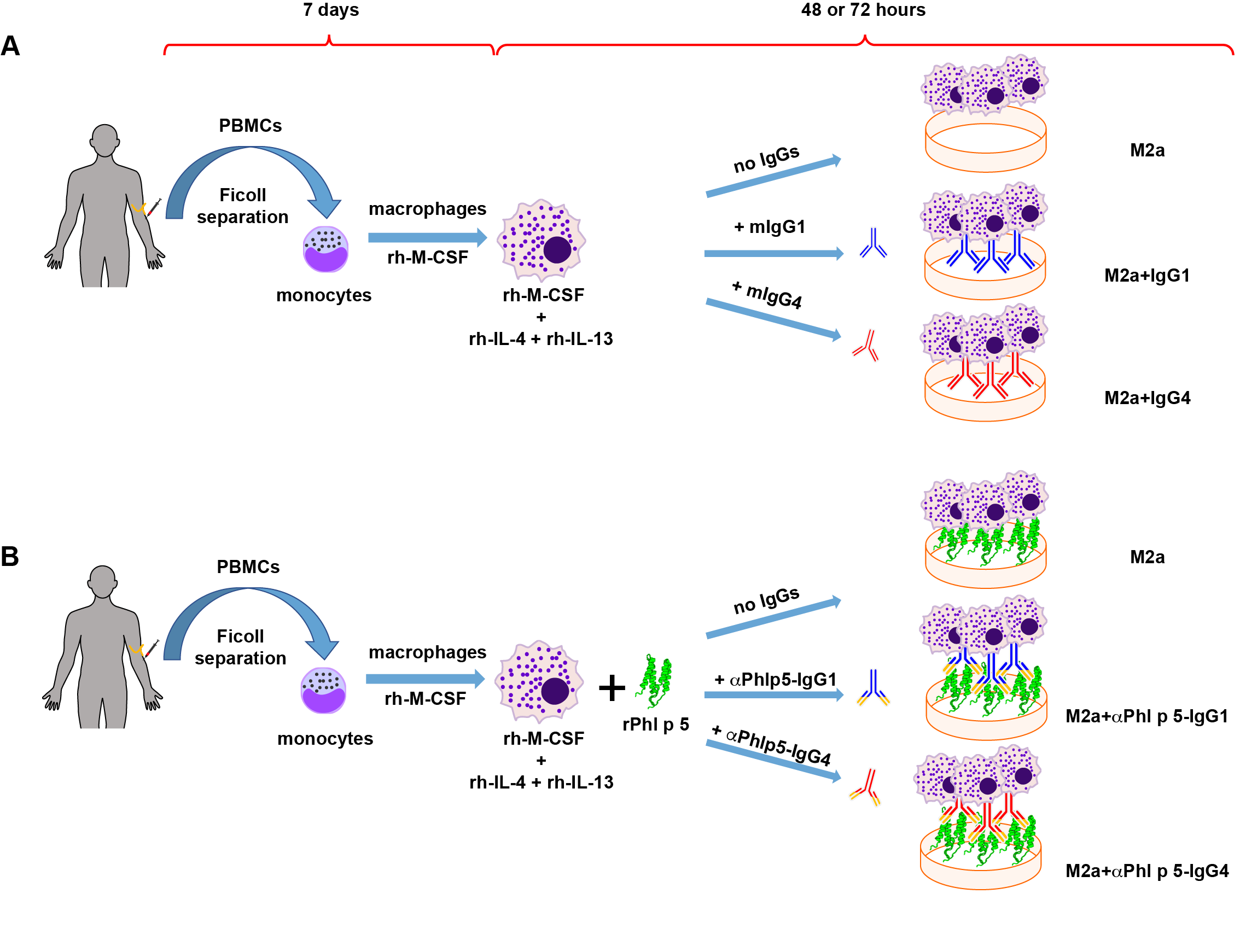


**Fig. S1:** Differentiation protocol of human peripheral blood monocytes to macrophages. Monocytes purified from human PBMCs were treated for 7 days with rh-M-CSF only, followed by activation with rh-M-CSF, rh-IL-4, rh-IL-13, and. the co-incubation with immune complexes consisting of either myeloma IgG1 (mIgG1) or myeloma IgG4 (mIgG4) (A), or recombinant human allergen-specific IgG1 or IgG4 (B) for 48-72h. **(A)** M2a cells were cultured in wells without, or with coated mIgG1 (blue; M2a+IgG1) or mIgG4 (red; M2a+IgG4). **(B)** Wells were coated with recombinant timothy grass pollen allergen Phl p 5 (green; rPhl p 5) and then were left untreated or incubated with anti-Phl p 5 specific IgG antibodies (αPhlp5-IgG1 or αPhlp5-IgG4). M2a cells were cultured in wells without, or with αPhlp5-IgG1 (blue; M2a+αPhl p 5-IgG1) or αPhlp5-IgG4 (red; M2a+ αPhl p 5-IgG4) antibodies.


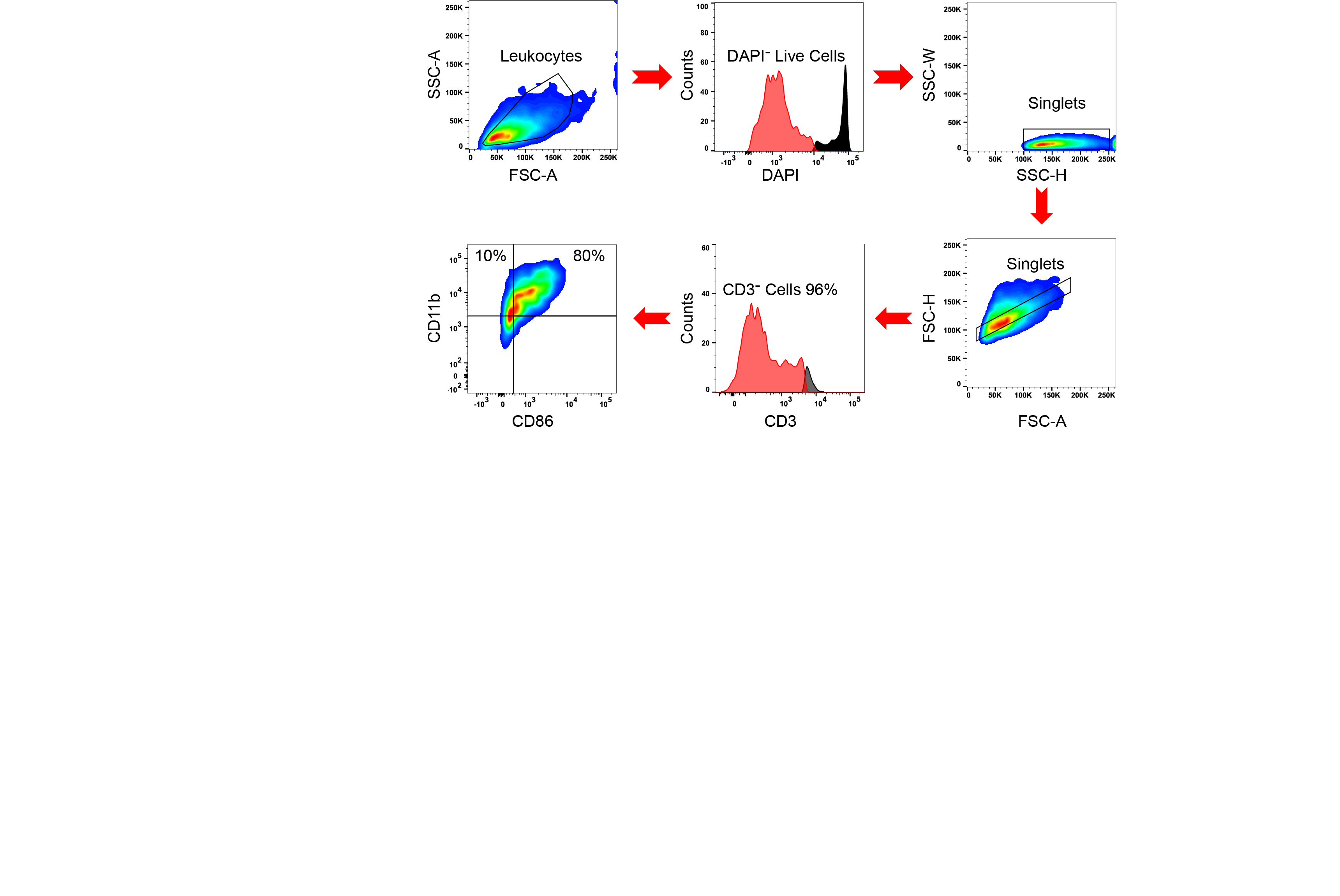


**Fig. S2:** Flow cytometry analysis of adherent cells after 3 days of differentiation. The cells were gated to discriminate live cells using dead staining (Live cells in red). The cells were then characterized as single cells using Side Scatter Width (SSC-W) and Side Scatter Height (SSC-H). Then the characterization was refined using the Forward Scatter Height (FSC-H) and Forward Scatter Area (FSC-A). Single cells were investigated using anti-hCD3, anti-hCD86 and anti-hCD11b antibodies. The percentage of CD3- cell (CD3^-^ Cells in red) is 96% ± 4%. CD3- cells were then analyzed to determine the purity of the monocytes. The percentage of CD3^-^CD86^+^CD11b^+^ is 80% ± 5%.


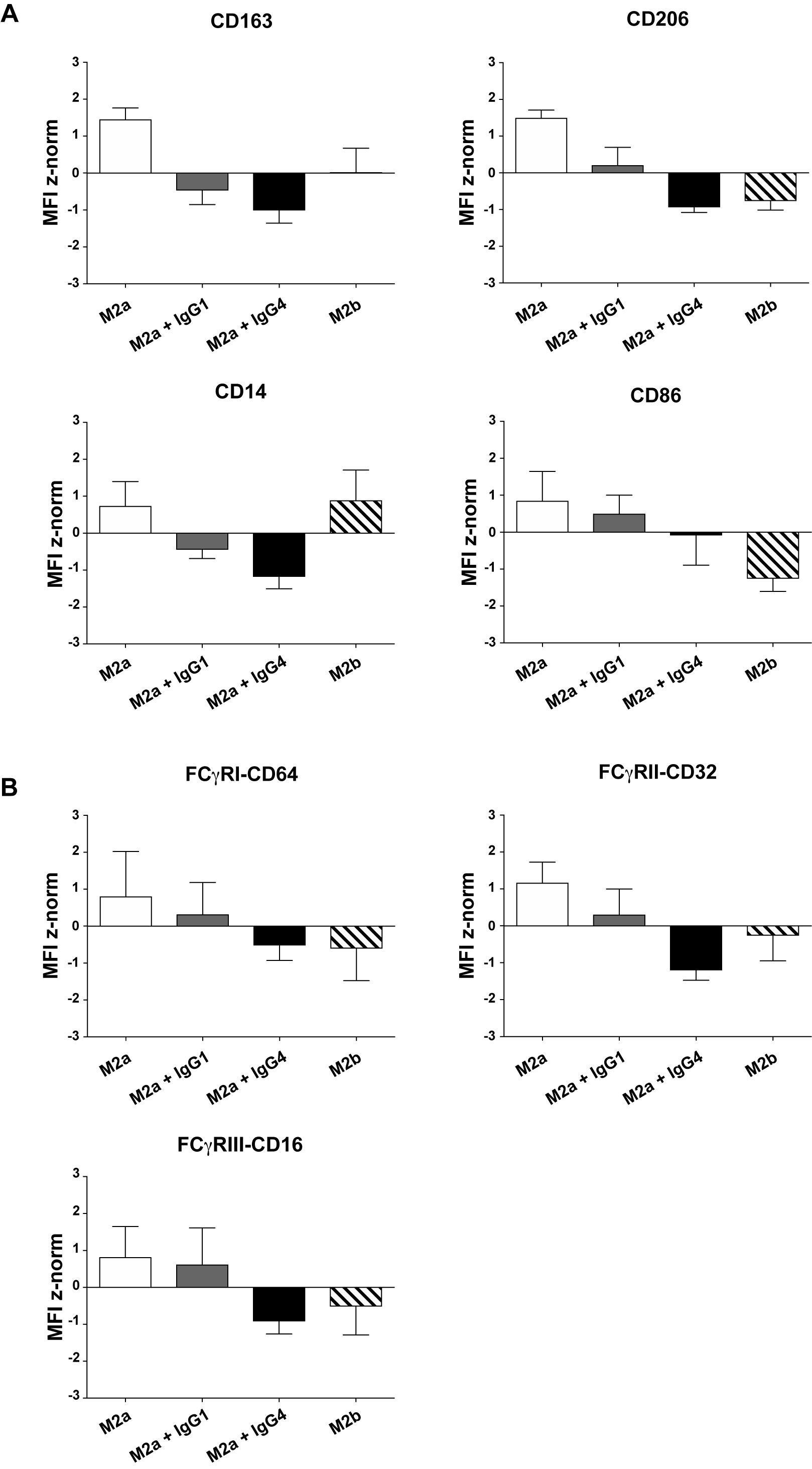


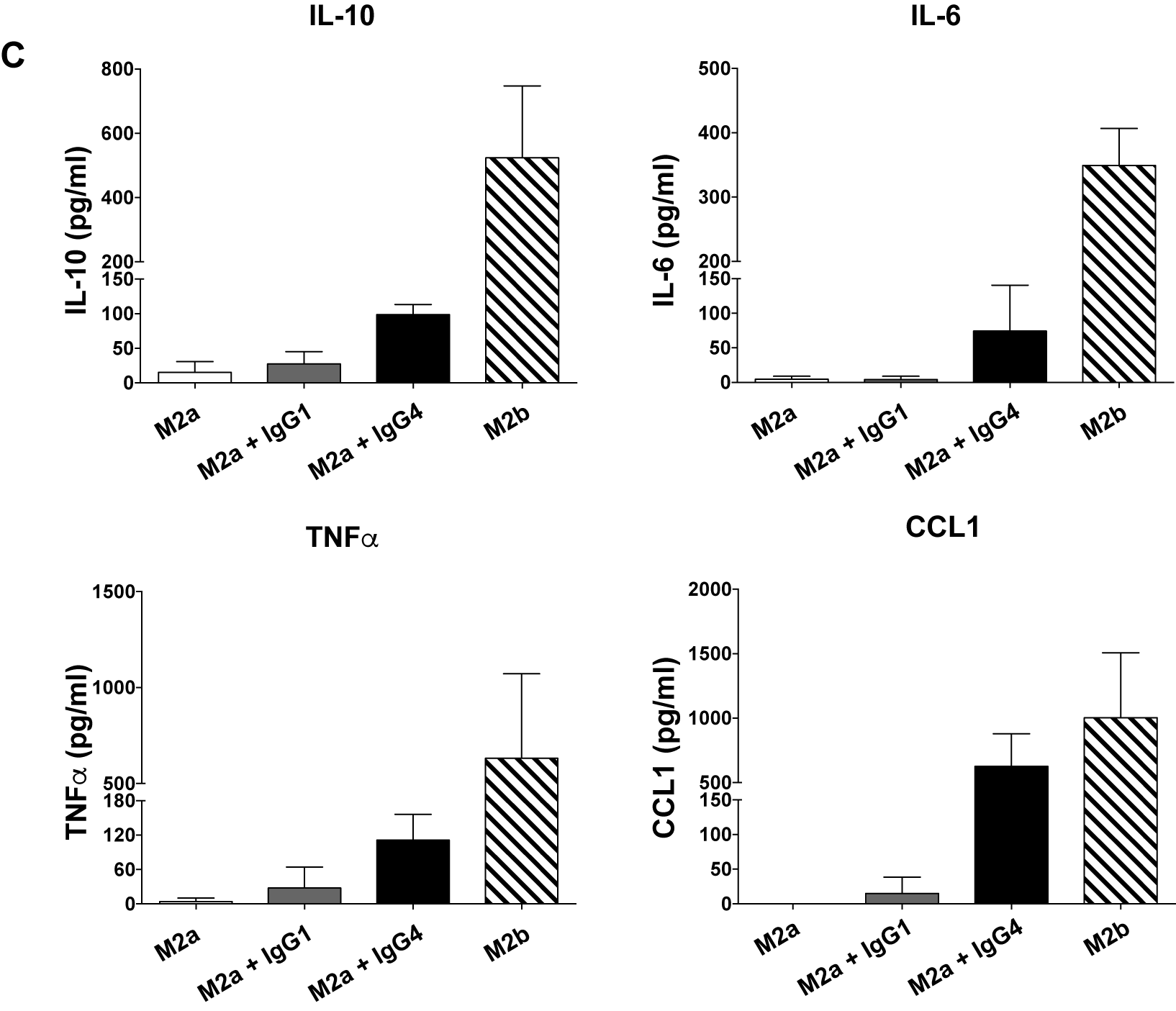


**Fig. S3:** Flow cytometry analysis of surface markers expressed after *in vitro* treatment for 72 h by M2a or M2b untreated cells or M2a treated with myeloma (m)IgG1 or mIgG4. MFI z-normalization was performed for each marker and each donor before the statistical validation. (A) MFI z-normalization of the MFI values for surface marker expressions of CD14, CD86, CD163 and CD206 were performed for each marker and each donor. The assay shown is representative of four independent experiments. (B) MFI z-normalization of the MFI value for surface marker expressions of FcγRs was performed for each marker and each donor. The assay shown is representative of three independent experiments. (C) ELISA analyses of IL-10, IL6, TNFα, CCL1 cytokine and chemokine secreted by M2a or M2b untreated cells or M2a treated with myeloma (m)IgG1 or mIgG4 macrophages polarized in vitro for 72 h. The assay shown is representative of three independent experiments. The y-axis represents the concentration (pg/ml) of the cytokines analyzed (± STDEV). White bars: M2a cells; grey: M2a cells +IgG1; dark: M2a cells +IgG4; obliquus filled bars: M2b cells.

**References**

1. Flicker S, Vrtala S, Steinberger P, et al. A human monoclonal IgE antibody defines a highly allergenic fragment of the major timothy grass pollen allergen, Phl p 5: molecular, immunological, and structural characterization of the epitope-containing domain. *J Immunol.* 2000;165(7):3849-3859.

2. Madritsch C, Flicker S, Scheiblhofer S, et al. Recombinant monoclonal human immunoglobulin E to investigate the allergenic activity of major grass pollen allergen Phl p 5. *Clin Exp Allergy.* 2011;41(2):270-280.
